# Conformation-dependent restraints for polynucleotides: the sugar moiety

**DOI:** 10.1093/nar/gkz1122

**Published:** 2019-12-04

**Authors:** Marcin Kowiel, Dariusz Brzezinski, Miroslaw Gilski, Mariusz Jaskolski

**Affiliations:** 1 Center for Biocrystallographic Research, Institute of Bioorganic Chemistry, Polish Academy of Sciences, Poznan 61-704, Poland; 2 Institute of Computing Science, Poznan University of Technology, Poznan 60-965, Poland; 3 Center for Artificial Intelligence and Machine Learning, Poznan University of Technology, Poznan 60-965, Poland; 4 Department of Crystallography, Faculty of Chemistry, A. Mickiewicz University, Poznan 61-614, Poland

## Abstract

Stereochemical restraints are commonly used to aid the refinement of macromolecular structures obtained by experimental methods at lower resolution. The standard restraint library for nucleic acids has not been updated for over two decades and needs revision. In this paper, geometrical restraints for nucleic acids sugars are derived using information from high-resolution crystal structures in the Cambridge Structural Database. In contrast to the existing restraints, this work shows that different parts of the sugar moiety form groups of covalent geometry dependent on various chemical and conformational factors, such as the type of ribose or the attached nucleobase, and ring puckering or rotamers of the glycosidic (χ) or side-chain (γ) torsion angles. Moreover, the geometry of the glycosidic link and the endocyclic ribose bond angles are functionally dependent on χ and sugar pucker amplitude (τ_m_), respectively. The proposed restraints have been positively validated against data from the Nucleic Acid Database, compared with an ultrahigh-resolution Z-DNA structure in the Protein Data Bank, and tested by re-refining hundreds of crystal structures in the Protein Data Bank. The conformation-dependent sugar restraints presented in this work are publicly available in REFMAC, PHENIX and SHELXL format through a dedicated RestraintLib web server with an API function.

## INTRODUCTION

Geometrical restraints for macromolecular structure, which *per se* are a treasury of our accumulated knowledge about molecular dimensions, are usually regarded as a necessary ingredient of crystal structure refinement at lower resolution, where the number of experimental data is insufficient to adequately define the numerous model parameters. However, restraints may also be needed even at very high resolution to fix the geometry of disordered fragments, which are not defined by diffraction, or to build likelihood functions which require not only accurate geometrical targets but also their error estimates. Apart from crystallography, stereochemical restraints are an essential component of NMR models and—with fast-growing importance—of cryo-EM models. Geometrical restraints are also of key importance in the area of computational modeling of macromolecular structure, which could be regarded as the ultimate case of modeling in the absence of direct experimental observations. Last but not least, reliable dictionaries of macromolecular geometry are necessary for proper validation of any structural models, especially in the Protein Data Bank (PDB) (1).

So far, the standard stereochemical restraint dictionaries for nucleic acids have been compiled by Taylor and Kennard (2) and later by Parkinson *et al.* (3). The Parkinson library was based on two detailed geometrical analyses, focused on the sugar-phosphate backbone (4) and on the nucleobase moiety (5). Recently, we have initiated a project aimed at reinvestigation of the nucleic acids restraints, motivated by the nearly tenfold expansion of the Cambridge Structural Database (CSD) (6), which stores crystal structures of organic molecules (currently over one million) serving as the source of accurate geometrical information. We were also inspired by the concept of conformation-dependent stereochemical libraries (CDL), introduced and successfully applied to proteins by Karplus *et al.* (7–9). Parenthetically, it is noted that the expansion of the PDB in the same 23-year period has been 40-fold, with a nearly 200-fold increase in the number of atomic-resolution (defined as 1.2 Å or better) structures. It has been shown more than once that the expansion of structural databases calls for periodic revisions of the existing restraint libraries (10).

We have defined our workflow differently than Parkinson *et al.*, dividing the polynucleotide macromolecule into the phosphodiester group (11), the nucleobase fragment (12) and the sugar moiety. The first two analyses have been already published, confirming, overall, the validity of the Parkinson library, but with notable exceptions and with improvements that have been made available for general use through a dedicated webservice called RestraintLib (http://achesym.ibch.poznan.pl/restraintlib/). As a side effect of our PO_4_ analysis, we were able to conclusively demonstrate that artificial intelligence and machine learning are now capable of discovering (without supervision) hidden geometrical patterns in structural data (11). In the work on nucleobases (12), we also investigated the limit of applicability of quantum-mechanical calculations and confirmed that nucleobase geometry derived from the CSD corresponds to Watson–Crick pairing regardless of the actual molecular environment of those bases.

**Figure 1.**
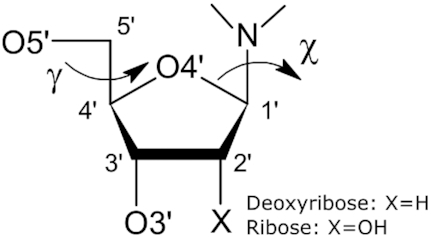
Covalent structure and atom numbering of the nucleoside sugar moiety discussed in this work.

In the present paper, the third in our series, we analyze the glycosidic moiety of nucleic acids chains, which also includes the glycosidic bond itself and its attachment to the nitrogenous base (Figure 1). This is the most difficult part of our analysis, as the sugar moiety is the most flexible one and indeed has molecular dimensions that are strongly dependent on ring puckering (13) and torsion angle conformations (14). We base our analysis on sugar fragments found in the CSD and discover statistically distinct subpopulations (groups) of bond lengths and angles, as well as functional relations linking covalent geometry with conformation. Based on the discovered groups, we propose a new, conformation-dependent set of restraints for the sugar moiety. Finally, the revised restraints are validated using ultrahigh-resolution data from the PDB, as well as massive re-refinement of PDB structures across a wide range of resolutions. The new glycosidic restraints are available through an updated version of our RestraintLib server, which can be integrated with existing software using a programmatic API function.

## MATERIALS AND METHODS

### Selection of CSD fragments

Sets of high-resolution structures containing ribose (R) or 2′-deoxyribose (D) attached to a purine (Pu) or pyrimidine (Py) base, were collected from the CSD version 5.40 update 2 using CONQUEST 2.01 (15). We analyzed the sugar moiety in conjunction with the bases in order to investigate the effect of base type (Pu, Py) on the bond lengths and angles in the proximity of the glycosidic link. We did not carry out separate analyses for the individual concrete nucleobases (A, C, G, T, U), as such queries resulted in too small sample sizes. The CSD Python API 2.1.0 (6) was used to compute geometrical parameters, which were later statistically analyzed, conformationally grouped, and averaged to yield the desired restraint targets and their standard deviations.

Only structures with *R* ≤ 8.5% and average estimated standard deviation (e.s.d.) of C–C bond lengths σ(C–C) < 0.01 Å were selected, based on the analysis presented in the next section. As in our previous studies from this series (11,12), we used a modified *Z*-score test (16) to identify and reject outliers. In this test, a data item ${x_i}$, in our case a bond distance or angle, is treated as an outlier if $| {{M_i}} | >\ 3.5$. ${M_i}$ is calculated as follows:

$$\begin{equation*}\ {M_i} = \frac{{0.6745\left( {{x_i} - \tilde{x}} \right)}}{{{\rm median}\left\{ {\left| {{x_i} - \tilde{x}} \right|} \right\}}}\ \end{equation*}$$

where $\tilde{x}$ denotes the median of the sample (R-Pu; D-Pu; R-Py or D-Py). When at least one parameter in a given CSD structure was tagged as an outlier, the entire structure was removed from the analysis. The effect of the various quality criteria on the number of rejected cases is illustrated in Supplementary Figure S1. In the final database of examples, there were 130 R-Pu sugar-base cases, 51 D-Pu cases, 84 R-Py cases and 167 D-Py cases. For comparison, the library of Parkinson *et al.* (3) was created using 80 ribose and 47 deoxyribose sugars, without taking into account whether or what type of base was attached. The CSD codes of the structures selected for this study are listed in Supplementary Table S1.

For terminal sugars, we queried the CSD separately with H atoms added explicitly to the O3′ or O5′ atoms. The resulting dataset contained 118 R-Pu sugar-base pairs, 37 D-Pu pairs, 75 R-Py pairs, 121 D-Py pairs for O3′ terminals, and 90 R-Pu pairs, 35 D-Pu pairs, 53 R-Py pairs, 113 D-Py pairs for O5′ terminals. The CSD codes of these structures are listed in Supplementary Table S2.

### CSD sampling methodology

The structure sampling criteria listed in the previous section were selected based on analyses of four quality indicators: (i) the *R*-factor, (ii) σ(C-C), (iii) all structures/only non-disordered structures and (iv) all structures/structures after outlier removal. To establish an optimal set of criteria, we analyzed two measures describing the sampled bond lengths and bond angles: the standard error of the mean (SEM), which assesses the uncertainty of the estimated mean value of a given geometrical parameter, and the standard deviation (STD), which quantifies the amount of variation (spread) in a sample. Our goal was to find such criteria that minimize both the SEM and standard deviation in the selected samples.

Supplementary Figure S2 shows how the average SEM (left) and standard deviation (right) of bond angles change when the *R* value (x-axis) increases from 4.5% to 10.0%. For all sugar-base pairs, the general trend is that the higher the *R*-factor, the smaller the SEM and the larger the standard deviation. The limit of *R*-factor ≤ 8.5% in our samples was selected as a compromise between the SEM and standard deviation, as for this threshold the SEM seems to level out and a higher threshold would only increase the standard deviation. Supplementary Figure S2 also shows that the outlier removal method presented in the previous section (Supplementary Figure S2, dash lines) significantly decreases both the SEM and standard deviation, and is, therefore, the most crucial quality factor. When analyzing samples with outlier removal, it can also be seen that using only non-disordered structures results in a much higher SEM. This stems from the fact that a structure may (usually) contain disorder outside of the queried sugar-base fragment, yet each eliminated structure decreases the sample size, negatively impacting the approximation of the mean. Finally, limiting the sampled structures to those with σ(C–C) < 0.01 Å, as in the procedure adopted by Parkinson *et al.* (3), results in a slightly smaller standard deviation of the sample (Supplementary Figure S2 right, panel columns). Similar observations were made for the SEM and standard deviation behavior for bond lengths (Supplementary Figure S3).

### Analytical tools for discovering consistent groups and functional relations

A number of statistical tests were used to verify whether subgroups of parameter values (bond lengths or angles) differ significantly from each other. Potential subgroups were defined by: (i) ring pucker (C2′-endo, C3′-endo, Other), (ii) χ torsion angle rotamer (syn, anti), (iii) γ torsion angle rotamer (trans, gauche+, gauche–), (iv) sugar type (ribose, 2′-deoxyribose), (v) base type (purine, pyrimidine). Analogously to Gelbin *et al.* (4), the C3′-endo state was defined for the pseudorotation angle *P* in the range 0° ≤ *P* ≤ 36° and the C2′-endo state for 144° ≤ *P* ≤ 190° (13), whereas Other denotes ribose ring puckering with any other values of *P*. The phase angle (*P*) and amplitude (τ_m_) of pseudorotation were calculated from the endocyclic ribose torsion angles using the method proposed in (17). The ranges of the glycosidic torsion angle were defined as |χ| ≤ 90° for syn and |χ| > 90° for anti, whereas the side chain rotamers were defined as gauche+ (γ = 60 ± 30°), gauche– (γ = −60 ± 30°), and trans (γ = 180 ± 30°).

Welch's two-sided *t*-test (18) was employed at the significance level α_t_ = 0.05 to determine whether two subgroups of parameter values form separate classes. We chose this test as it does not assume equal population variance. When several subgroups were found to be statistically different from each other, the restraints were calculated separately for each subgroup. If more than one set of subgroups was statistically significant (e.g. for sugar type and ring conformation), we verified whether the majority of subgroups defined using pairs of these variables (e.g., ribose C2′-endo) were also significant. If pairs of variables were significant, we based the restraints on variable combinations. Otherwise, we selected the variable with the smallest *p*-value.

Apart from determining statistically significant subgroups, we also looked for functional relationships between bond lengths/angles and three continuous conformational variables: the glycosidic torsion angle χ, the side chain torsion angle γ, and sugar pucker amplitude τ_m_. Spearman rank correlation (19) was used to establish whether there exists a potential functional relationship between a bond length/angle and a variable. To ensure that the functional relationship is strong and statistically significant, we only investigated potential relationships that had an absolute value of observed Spearman correlation |ρ| >0.5 and were determined to be statistically different from zero at the significance level α_S_ = 0.05. As was done with the parameter groups above, if functional relationships were found in subgroups, we checked whether the majority of functional dependencies in a subgroup were statistically significant. We chose Spearman correlation for this task as it is a non-parametric measure and is capable of detecting linear as well as non-linear relationships.

For each detected functional relation, a regression function was determined using Gaussian Process Regression (GPR) (20). We chose GPR because it takes into account sample noise, estimates not only the mean value but also provides the standard deviation of the predicted value, and it can handle periodic functions, which is needed when the independent variable is a cyclic torsion angle. For bond lengths/angles with non-linear dependence on torsion angles, we tested GPR with an exp-sine-squared kernel with 180° and 360° periods combined with a white noise kernel with noise levels ranging from 10^−7^ [Å/°] to 10^7^ [Å/°]. For linear relations, we tested Bayesian Ridge Regression, which is equivalent to GPR with a linear kernel. In both cases, the data were mean-normalized prior to fitting and the final functions were selected by maximizing their log marginal likelihood.

All computational experiments were scripted in Python 2.7 using the scipy (21) and scikit-learn (22) libraries.

## RESULTS AND DISCUSSION

### Geometrical characteristics of the sugar moiety

Compared to the phosphodiester and nucleobase fragments studied in our previous papers from this series (4,5), the sugar moiety of nucleic acids is much more complex and flexible. Whereas the analysis of phosphodiester fragments revealed coherent groups of conformations and the bases were found to have fixed geometries, it is quite clear that particular bond lengths and angles of the glycosidic moiety depend on a number of variables, including conformational parameters. Being entangled between the phosphodiester and the base moieties, different parts of the sugar unit depend in different degree on the type and geometry of these two neighbors. Therefore, each bond length and angle of the glycosidic fragment has to be studied separately in search of consistent groups and functional relations.

#### Bond lengths

**Figure 2.**
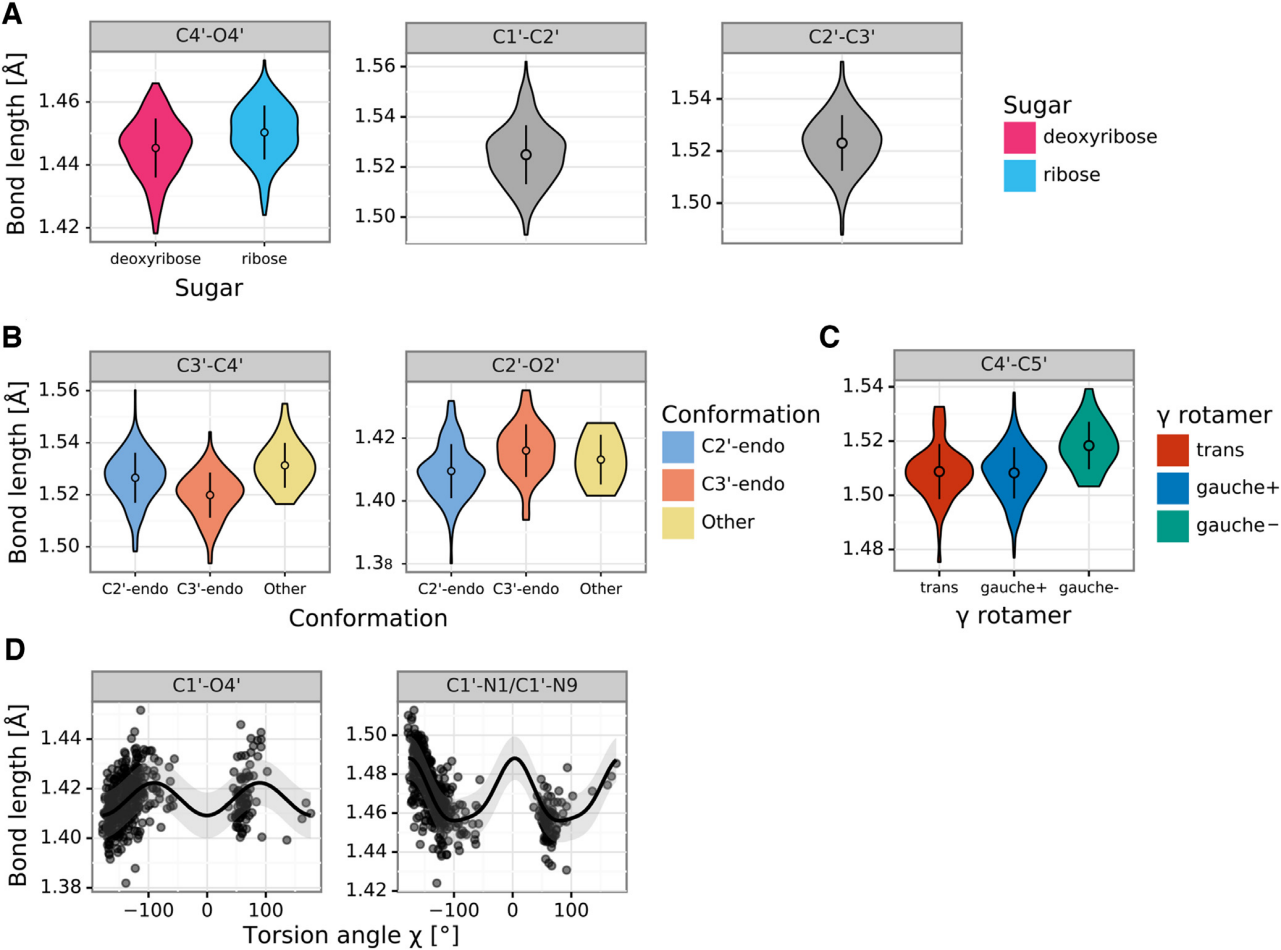
Bond length distributions in sugar fragments retrieved from the CSD. (**A**) Violin plots of bond lengths (panels) that are independent of any factors (gray) or depend on sugar type (color). (**B**) Violin plots of bond lengths that depend on ring conformation (color). (**C**) Violin plots of bond lengths that depend on the rotamer of torsion angle γ (color). (**D**) Scatter plots and regression lines for bond lengths that functionally depend on the glycosidic torsion angle χ (x-axis). A violin plot presents the shape of a distribution, as well as the mean value (point) and standard deviation (error bar). Scatter plots include the mean (line) and standard deviation (semi-transparent band) calculated by Gaussian Process Regression.

Figure 2 presents the four main sets of relations discovered for bond lengths. The first and second set (Figure 2A, B) include sugar ring bonds that depend on ring pucker conformation (C2′-endo/C3′-endo/Other), sugar type (ribose/2′-deoxyribose) or have fixed lengths. The differences in group mean values between groups for a given bond length are up to 0.01 Å (e.g. C3′–C4′, C2′-endo compared to C3′-endo). The bond distances in these sets owe their variability to the flexibility of the sugar ring. It is worth noting that C1′–C2′ and C2′–C3′ were the only two bond lengths that were normally distributed according to the Shapiro–Wilk test (23), and that is why they were not divided into groups.

The next set (Figure 2C) contains the side chain C4′–C5′ bond, which depends on the rotamer of the torsion angle γ (trans, gauche+, gauche–). The differences in mean bond lengths are similar to those found in sugar ring bonds, and are also at the level of 0.01 Å. Once again, the relation in this case is a local one, as the C4′–C5′ bond is directly part of the torsion angle γ.

The last set of bond relations (Figure 2D) shows that the glycosidic bond length is a function of χ. The effect of this relation is very strong, with differences in the bond length of C1′–N1/C1′–N9 reaching 0.04 Å. According to the GPR analysis, the glycosidic bond (C1′–N1/C1′–N9) length is a sinusoidal-like function of χ with a period of 180° (Supplementary Figure S4). Interestingly, it can be noticed that the extreme values of the regression function lie close to χ = ±90°. Indeed, at |χ| = 90° the attached base is in a favorable geometrical orientation relative to the sugar ring and any rotation away from this position towards the syn or anti conformation will force the glycosidic link to lengthen.

#### Bond angles

**Figure 3.**
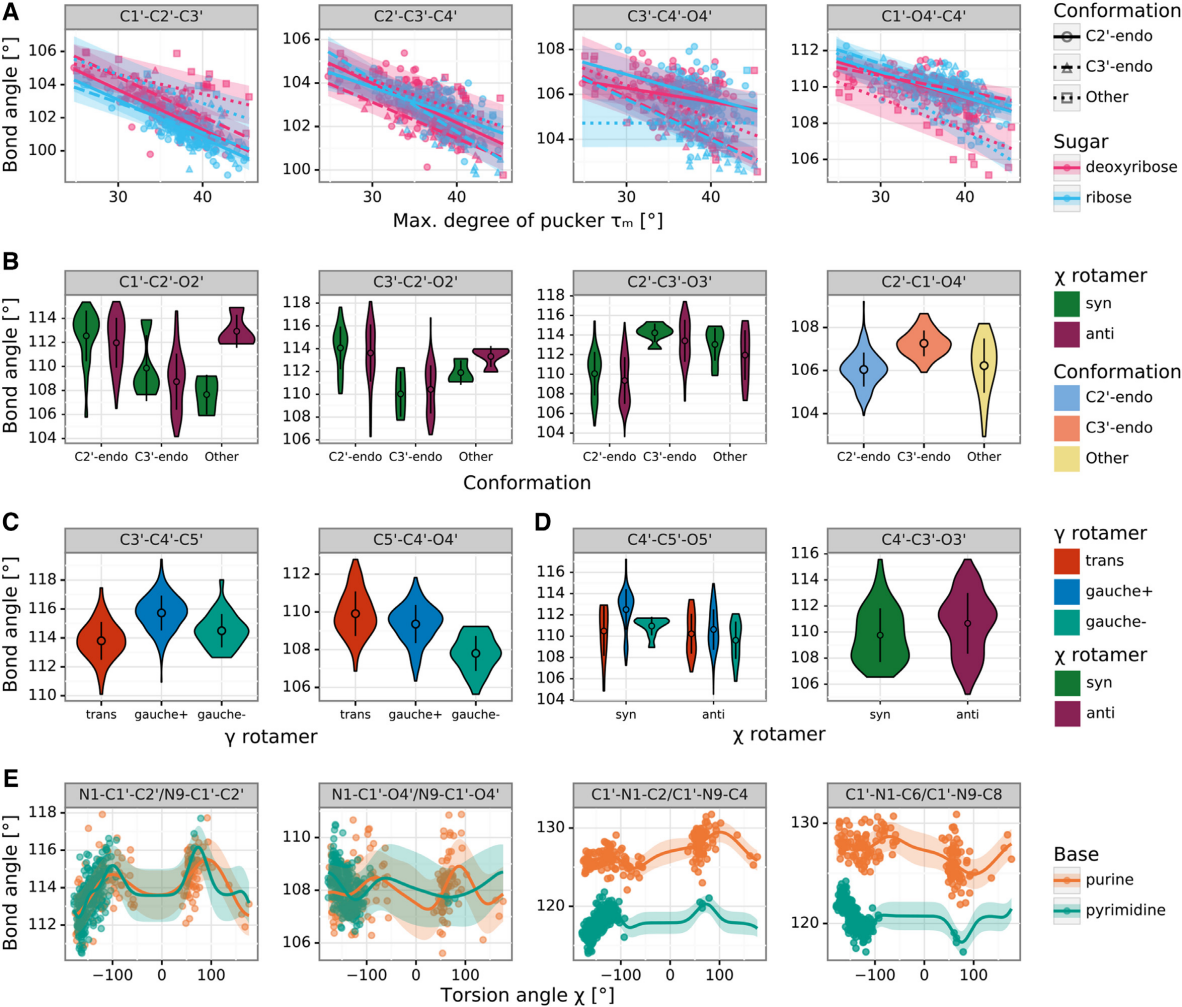
Bond angle distributions in sugar fragments retrieved from the CSD. (**A**) Scatter plots and regression lines for angles (panels) that functionally depend on sugar pucker amplitude τ_m_ (x-axis), ring conformation (color), and sugar type (shape). (**B**) Violin plots of angles that depend on ring conformation (x-axis) and the glycosidic torsion angle χ (color). (**C**) Violin plots of angles that depend on the rotamer of torsion angle γ (color). (**D**) Violin plots of angles (panels) that depend on the rotamer of the glycosidic torsion angle χ (color) and/or torsion angle γ (x-axis). (**E**) Scatter plots and regression lines for angles that functionally depend on the glycosidic torsion angle χ (x-axis) and base type (color). A violin plot presents the shape of a distribution, as well as the mean value (point) and standard deviation (error bar). Scatter plots include the mean (line) and standard error (semi-transparent band) calculated by Gaussian Process Regression.

Figure 3 presents five sets of relations discovered for bond angles. The relations in the first set (Figure 3A) affect the endocyclic angles at the ribose ring, which are linearly dependent on the sugar pucker amplitude τ_m_, and discretely on the type of ring pucker and type of sugar. Generally, the larger the maximum degree of pucker, the smaller the endocyclic angles, with differences ranging from 2° to 5°. The only exception from this rule is the C3′–C4′–O4′ angle of ribose with Other ring conformation; in this case, according to the current CSD sample there is no functional relationship and the angle seems to be constant.

The next sets of relations show angles grouped according to ring pucker and χ rotamer (anti, syn) (Figure 3B), as well as angles at the C5′ side chain, which cluster according to γ and χ (Figure 3C, D). The biggest differences in these sets (∼4°) can be found for angles involving the C2′ and C3′ atoms, which form the pivotal element differentiating the C2′-endo and C3′-endo types of (deoxy)ribose ring conformations. The remaining relations account for angular differences at the level of 1–2°.

The last set (Figure 3E) consists of angles at the glycosidic bond, which show functional dependence on χ and on base type. This relation is a consequence of the strong functional dependence found for the glycosidic bond length, and results in differences up to the level of 5°. The best fitted regression functions are periodic but not strictly sinusoidal. Moreover, we can see extreme values around χ = ±90°, but there are clear differences between the syn and anti conformations, which introduce asymmetry to this functional relationship.

### The proposed restraints

Tables 1 and 2 present the mean values with standard deviations of the proposed sugar restraints for bond lengths and angles, respectively. Letters in the upper left corners of both tables correspond to parameter sets presented in Figures 2 and 3. For comparison, Tables 1–2 also present the mean values listed in the compilation by Parkinson *et al.* (3).

**Table 1.** CSD-derived mean values and standard deviations (in parentheses, in units of the last significant digit of the mean value) for sugar bond lengths (in Å)

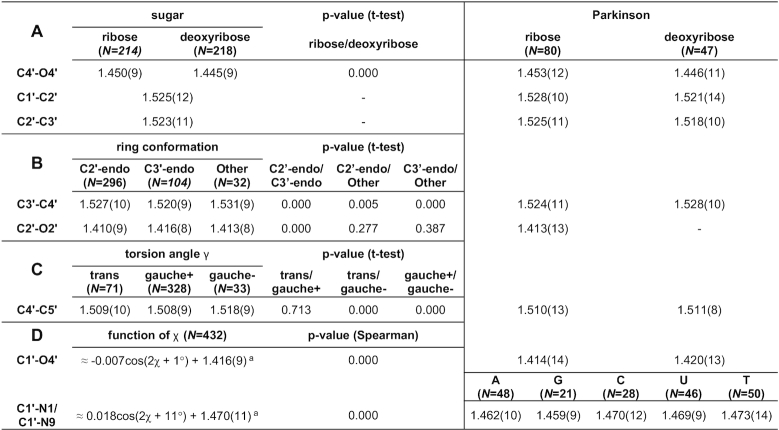

*N* is the number of cases used to compute a given restraint. A, G, C, U, T, denote adenine, guanine, cytosine, uracil, and thymine. Columns labeled Parkinson report reference values from (3). ^a^Function definitions presented for bonds C1′-N1/C1′-N9 and C1′-O4′ are approximations obtained using least-squares regression with formula *a·cos(x + b) + c* for discussion purposes; the Gaussian Process Regression used to obtain the actual restraints is based on example distance and its results cannot be presented in a single formula.

**Table 2.** CSD-derived mean values and standard deviations (in parentheses, in units of the last significant digit of the mean value) for sugar bond angles (in °)

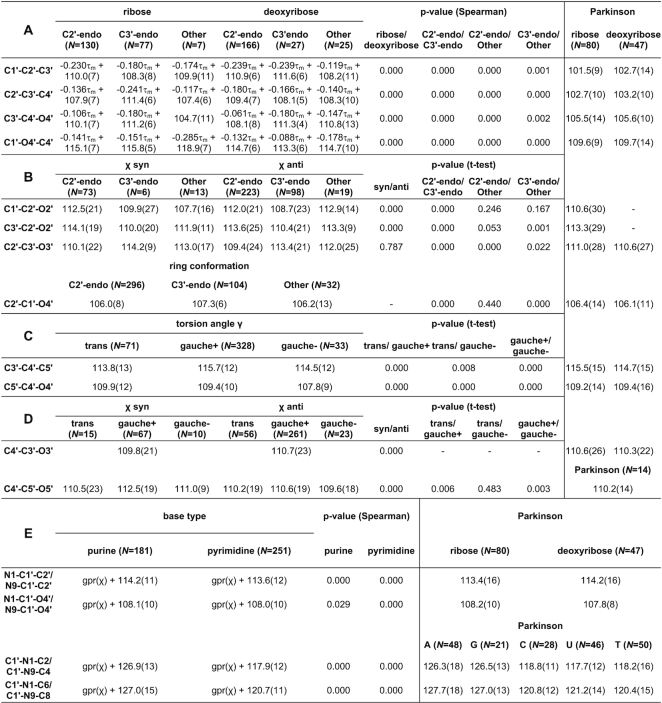

*N* is the number of cases used to compute a given restraint. A, G, C, U, T, denote adenine, guanine, cytosine, uracil, and thymine. Columns labeled Parkinson report reference values from (3). gpr(_X_) denotes a Gaussian Process Regression function of _X_, with its mean shift given as an added coefficient.

The bond sets containing sugar ring bonds (Table 1A, B) have restraints divided according to sugar type and ring conformation. The values from the Parkinson library (3), which distinguished only between ribose and deoxyribose, have mostly higher standard deviations and correspond mainly to the C2′-endo conformation, which is mostly found in B-type duplexes. On the other hand, the restraints proposed in the present study show differences in bond lengths for different ring conformations that reach 0.01 Å (e.g. C3′–C4′). The work of Gelbin *et al.* (4) studied the possibility of differentiating between C2′-endo and C3′-endo conformations, but most (13 out of 19) of their differences in bond lengths were found to be statistically insignificant, leaving the final Parkinson library without this differentiation (3). In the present study, the approach of defining restraint groups for each bond separately allowed us to separate C2′-endo and C3′-endo conformations that were statistically different according to Welch's *t*-test at the significance level of α_t_ = 0.05, while grouping the remaining bonds according to other criteria. Differences between the C2′/C3′-endo and Other conformations were not always statistically significant, but the Other category allowed us to take into account the full spectrum of possible pseudorotation values.

With regard to the C4′–C5′ bond (Table 1C), the Parkinson library shows practically identical values for ribose and deoxyribose, whereas the γ torsion angle categorization proposed here shows statistically significant differences between gauche*–* and gauche+/trans conformations. The values from the Parkinson compilation seem to be closer to the more popular gauche+/trans conformations but have higher standard deviations. The C4′–C5′ bond illustrates the fact that a simple distinction between ribose and 2′-deoxyribose may be insufficient to capture the flexibility of the sugar moiety.

Restraints in Table 1D show approximations of the functional relations for the glycosidic bond. Although the GPR algorithm uses an instance-based kernel method which depends on the CSD training examples, the shape of the regression curve for glycosidic bonds is practically sinusoidally dependent on χ with a period of 180°. Therefore, in Table 1D we decided to show formulas that one would obtain using least-squares regression for a function of the form *a·*cos*(χ*/2 *+ b) + c*. This approximation shows the amplitude (*a*), phase shift (*b*) and mean/vertical shift (*c*) of the fitted cosine function. The mean values of so defined functions (*c*) are close to the values presented in the Parkinson library, however, the amplitude of changes can be from 0.007 Å to 0.018 Å. This means that the actual difference between the minimum and maximum bond length (2*·a*) can be from 0.014 Å to as much as 0.036 Å, depending on the value of the torsion angle χ. This shows the magnitude of flexibility at the glycosidic link, which was not clearly seen in the previous studies.

The endocyclic angles at the ribose ring (Table 2A) are statistically significantly dependent on the sugar pucker amplitude τ_m_ within groups defined by sugar type and ring conformation. The function is a linear one and can, therefore, be presented in Table 2A in exact form as *a·*τ_m_*+ b*. All the functions found in this class have a negative slope *a*, meaning that the higher the value of τ_m_, the smaller the endocyclic bond angles. Seeing that the slope of these functions is between −0.088 and −0.241, the possible 20° change in τ_m_ can result in a change of up to 4.8° for a given valence angle. The restraint targets in the Parkinson library do not take this τ_m_ dependence into account and, therefore, have higher standard deviations.

The next sets of sugar angles (Table 2B–D) are defined by groups rather than functions. Angles at the C2′–O2′/C3′–O3′ groups (Table 2B) depend on the χ angle and ring conformation. Interestingly, the glycosidic torsion angle χ also influences these angles, probably due to the fact that χ expresses the degree of ‘overlap’ between the sugar moiety and the attached base. The Parkinson library only distinguishes between ribose and deoxyribose versions of these angles, and misses the differences highlighted in our study. Furthermore, the valence angles at the C5′ atom (Table 2C, D) seem to depend on the torsion angles γ and χ. It is worth noting that the mean values of these valence angles in sugars with γ gauche– and χ syn conformations differ significantly (by ∼2°) from the values presented in the compilation of Parkinson *et al.* (3).

Finally, the functional relationships between χ and the angles N1/N9–C1′–C2′, N1/N9–C1′–O4′, C1′–N1/N9–C2/C4, C1′–N1/N9–C6/C8 (Table 2E) are not strictly sinusoidal and cannot be presented by a simple formula. Although the mean values of these functions correspond well with the values presented by Parkinson *et al.* (3), the Parkinson library does not take into account the functional dependence on χ, and this omission results in higher standard deviations.

Even though the restraint categorization proposed in this work is far more detailed than those presented by Parkinson *et al.* (3) and Gelbin *et al.* (4), the sample sizes of parameter groups in our study (denoted as *N* in Tables 1 and 2) are larger. This is one of the reasons why, compared to the previous studies, more relationships were discovered and could be confirmed to be statistically significant. Nevertheless, as the CSD continues to grow, it would be beneficial to periodically reiterate such analyses to ensure that the mean values and standard deviations used as restraints are as accurate as possible.

### **Terminal O5**′**/O3**′ **sugars**

The above set of restraints for polynucleotide sugar links was complemented with a separate analysis of terminal sugars. Since the proposed restraints are defined for each bond and angle separately, the analysis of terminal sugars involved only parameters directly associated with the O3′ and O5′ atoms, i.e. C3′–O3′, C5′–O5′, C2′–C3′–O3′, C4′–C3′–O3′, C4′–C5′–O5′. Table 3 presents the restraints proposed for these terminal bonds and angles.

**Table 3.** CSD-derived mean values and standard deviations (in parentheses, in units of the last significant digit of the mean value) for terminal sugar bond lengths (in Å) and angles (in °)

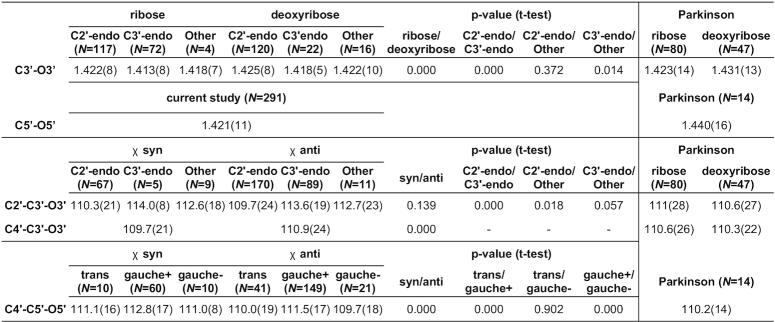

*N* is the number of cases used to compute a given restraint. Columns labeled Parkinson report reference values from (3).

The differences between the restraints for non-terminal (presented in our previous study of the phosphodiester group from this series (11)) and terminal (Table 3) C3′–O3′/C5′–O5′ bond lengths are quite substantial. Whereas non-terminal C3′–O3′ and C5′–O5′ bonds have (depending on PO_4_ category) bond lengths between 1.422–1.438 Å and 1.428–1.437 Å, respectively, their terminal counterparts are over 0.010 Å shorter (1.414–1.422 and 1.421 Å). Compared with the values listed in the Parkinson library, the differences are even bigger, reaching 0.019 Å for C5′–O5′. Indeed, the C5′–O5′ bond is expected to change its length significantly, reflecting the change of its chemical status (phosphoester or terminal hydroxyl). This shows the importance of taking into account also the border cases when compiling a comprehensive library of geometric restraints.

On the other hand, the C2′–C3′–O3′, C4′–C3′–O3′, C4′–C5′–O5′ angles at the terminal sugar hydroxyls are fairly similar to those of non-terminal sugars, with differences between these two sets ranging from 0.1° to 0.9°. The largest difference was found for C4′–C5′–O5′ in sugars with χ anti and γ gauche+ conformations, where the non-terminal/terminal sugars have mean values for this angle equal to 110.6°/111.5°. The standard deviations of the parameters calculated for terminal sugars were usually identical or marginally lower than those calculated for non-terminal sugars.

### Validation of the restraints using high resolution PDB and NDB data

We validated the proposed restraints for the sugar moieties of nucleic acids in two tests using high quality crystal structures reported in the PDB and in the Nucleic Acid Database (NDB) (24). First, we assessed the new restraints using an ultrahigh-resolution (0.55 Å) PDB structure 3P4J of Z-DNA refined without stereochemical restraints (25). In the second test, we computed absolute and RMS differences between the new restraints and bond lengths/angles of high-quality NDB structures with crystallographic resolution *d*_min_ ≤ 1 Å and *R* ≤ 10%.

When the sugar units of the 3P4J model are compared first with Parkinson restraints and then with our library, the RMSD(angles) value drops from 1.49° to 1.09° and RMSD(bonds) drops from 0.0083 Å to 0.0077 Å. The importance of positive validation against 3P4J lies in the fact that the 3P4J model was refined by the method of least-squares without *any* geometrical restraints (24), and is, therefore, not biased by any *a priori* geometrical assumptions.

The second validation involved 22 high-quality oligonucleotide structures from the NDB, containing altogether 312 sugar units; the PDB codes of those structures are listed in Supplementary Table S3. For most of those structures, it is not possible to determine whether they were refined with or without restraints. Four out of the 22 NDB structures were deposited prior to Parkinson's publication (3), and it is therefore reasonable to assume that they were refined without any restraints. For the remaining structures that were refined under restraint control (not always obvious), the Parkinson dictionary was most likely used. However, refinement against very high resolution data is usually able to override, at least partially, the information injected to the system by geometrical restraints. Nevertheless, to minimize potential bias introduced by restraints, we took into account only well-ordered fragments of those structures.

**Figure 4.**
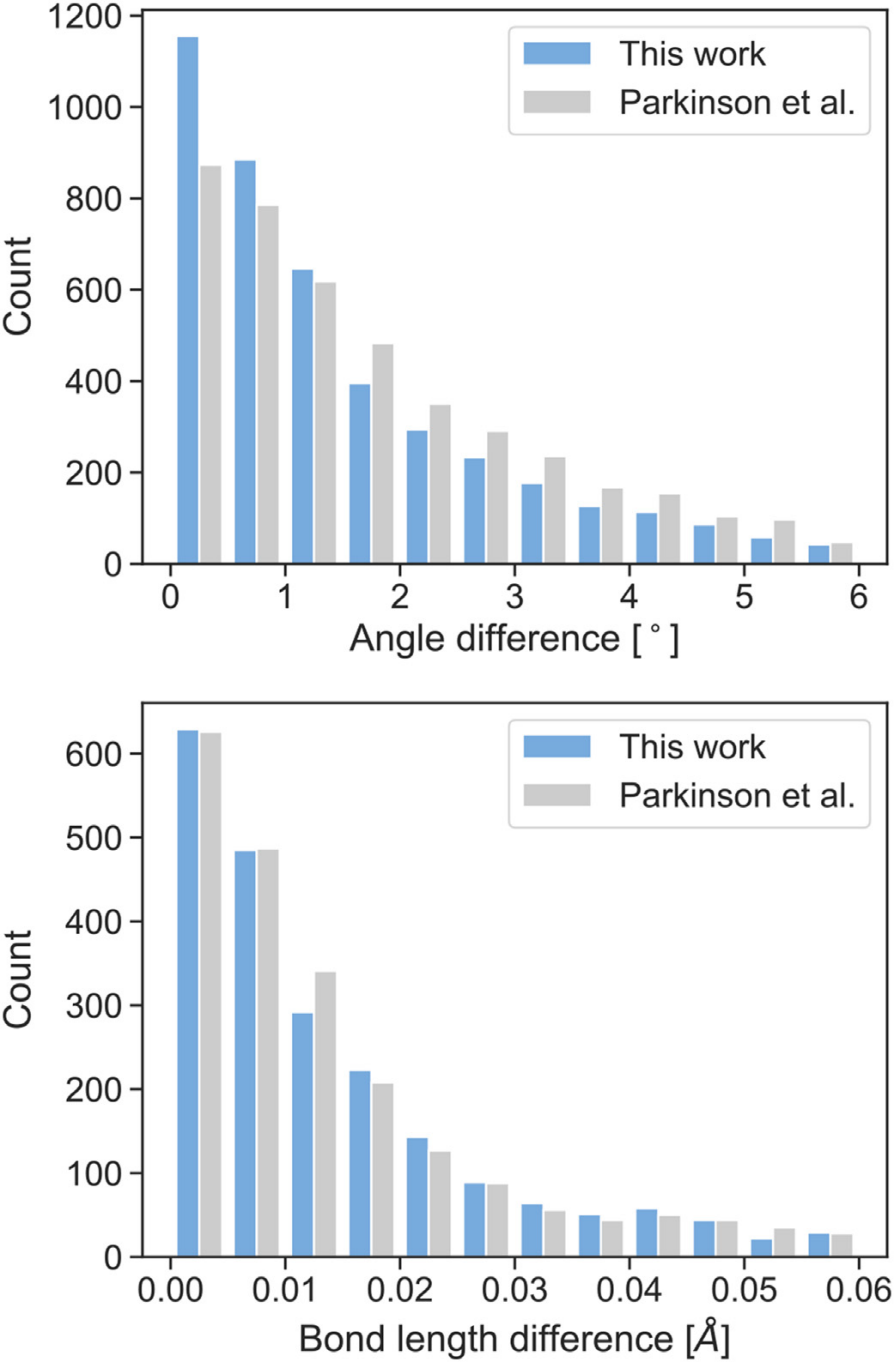
Histograms comparing the absolute differences in angles (top) and bond lengths (bottom) between the NDB values and the restraint targets compiled by Parkinson *et al.* (gray) and proposed in this paper (blue).

We compared in a histogram the absolute differences in bond angles (and separately in bond distances) between the model parameters and reference values from our work or from the Parkinson library (3). The histograms in Figure 4 show that the proposed angle restraints are superior to those reported by Parkinson *et al.*, whereas bond restraints from both libraries are of comparable quality. Quantifying this quality with RMSD, one gets RMSD(bonds) of 0.0272/0.0274 Å and RMSD(angles) of 2.67/2.45° for Parkinson/present work. According to the Wilcoxon signed-rank test (26), there is no significant difference between the accuracy of the bond lengths proposed by Parkinson *et al.* and in this work (*p*-value = 0.110), while the proposed sugar angle restraints are significantly better than those in the Parkinson library (*p*-value < 0.001).

The results confirm that the covalent geometry of nucleic acids is correlated with conformation. Similarly to our previous work (11), the geometric variations are much more prominent in bond angles than in bond lengths. Judging from the RMSD scores for structure 3P4J and the NDB data, any further improvement of restraints for bond lengths may be much harder to achieve than for angle restraints.

### Practical examples

The last step of validating our conformation-dependent sugar restraints involved refinement of various crystal structures using the proposed restraints. For this purpose, we used the 1.95 Å resolution protein-DNA complex with the PDB code 2HAN (27), the 2.70 Å RNA structure 429D (28), and the 0.97 Å DNA structure 4R15 (29). The structures were re-refined using 30 iterations of REFMAC version 5.8.0235 (30,31), 5 macrocycles of PHENIX version 1.17.1 (32), and 10 cycles of CGLS/L.S. in SHELXL (33), respectively. To ensure that the original and present results are comparable, we first re-refined the structures using the restraints built in the current versions of the programs or straight Parkinson library in the case of SHELXL, and next repeated the process using the proposed sugar restraints.

With external restraints, the refinement results strongly depend on tunable weight parameters. In REFMAC, these parameters are *w*, *w*_ext_ and *κ*, where *w* weights the contribution of the experimental data, *w*_ext_ adjusts the weights of the external restraints relative to other geometry components, and *κ* is the Geman–McClure robust estimation function parameter (34). Similarly, in PHENIX the contribution of the experimental data can be scaled by parameter *wxc*. In SHELXL, the situation is most straightforward because the geometrical restraints are weighted by 1/σ^2^, where σ is the standard deviation attached to each restraint target. To assess the quality of re-refinement with and without external restraints, we first found the parameter values that gave an RMSD(angles) of ∼1.80°, and then compared the *R*, *R*_free_, and RMSD values. The results of these validating experiments are presented in Table 4. They show that, in comparison with standard restraints, the new restraints can lead to a model that has the same or better stereochemical quality, but shows better agreement with the experimental data. In the case of the 2HAN structure, depending on REFMAC parameterization, *R*/*R*_free_ can be improved from 18.59/21.74% to 18.07/21.55%. Taking into account that the discussed new restraints affect only 40 sugar moieties (318 atoms) in a 2390-atom protein–DNA structure, the improvement can be considered substantial. A detailed analysis of how tuning the *w*, *w_ext_* parameters affects the re-refinement quality is presented in Supplementary Figure S5.

**Table 4.** *R*, *R*_free_, RMSD(bonds) and RMSD(angles) for the 2HAN/429D/4R15 models deposited in the PDB and re-refined using, respectively, REFMAC/PHENIX/SHELXL with default restraints and with restraints proposed in this work.

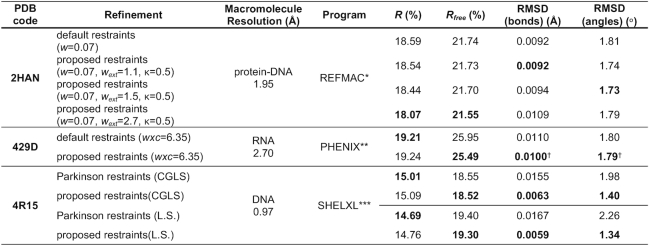

* REFMAC version 5.8.0253; ** PHENIX version 1.17.1; *** SHELXL version 2018/3; ^†^ PHENIX calculates RMSD values only for bonds/angles without external restraints. The marked RMSD values were calculated by an external script that compares the model against the restraint targets actually used during refinement.

In the case of the RNA structure 429D refined using PHENIX, *R*_free_ can be improved from 25.95% to 25.37%, without compromising the RMSD values. We note that PHENIX calculates RMSD values only for bonds and angles without external restraints. In practice, this feature of PHENIX suggests that the weight of external restraints (*wxc*) should be manually tuned rather than left to the default optimizer. Moreover, it might also be recommended to manually monitor (using an external utility) the evolution of the RMSD values (calculated against the actual restraint targets) during the course of the refinement. Therefore, in Table 4 we present the RMSD values that were obtained by comparing the model against all the actually applied restraints (external for sugars, default for the remaining moieties).

The DNA structure 4R15 was refined in SHELXL using the sugar restraints proposed by Parkinson *et al.* (3) as well as those recommended in this paper. Since SHELXL weights restraints according to the standard deviation attached to each restraint target, there is no simple way to tune RMSD(angles) at the 1.80° level. Thus in this example, the difference in quality is seen mainly in RMSD values for bonds/angles, which improved from 0.0155 Å/1.98° to 0.0063 Å/1.40° upon CGLS refinement, and from 0.0167 Å/2.26° to 0.0059 Å/1.34° when full-matrix L.S. refinement was performed (Table 4). The improvement is significant and shows that the proposed sugar restraints are more consistent with the physical structures. Additionally, both the CGLS and L.S. refinements converged with a slightly improved *R_free_* when the proposed restraints were used, underlying the fact that the geometrical model improvement is *not* offset by degradation of consistency with the experimental diffraction data.

We note that the presented examples of re-refining previously deposited models are not the targeted application of the proposed restraints. Typically, the proposed restraints would be used to aid the earlier stages of model building and preliminary structure refinements. In such situations, it would be worthwhile to regenerate the external restraints after each refinement run, as bonds and angles may change their conformational group assignment or functional relation results. Therefore, in addition to the examples presented above, we also used the proposed restraints for the refinement of a novel 1.6 Å RNA (40 nucleosides) crystal structure (unpublished results) in PHENIX (32). During the refinement, we monitored the percentage of the restraint targets that changed their conformational group with each iteration. As illustrated in Supplementary Figure S6, at each refinement step, up to ∼10% restraint groups changed, depending on the amount of modifications (e.g. alternative conformations) introduced to the model between the refinement rounds. The average change in angle restraint target oscillated between 0° and 0.15°, whereas distance restraints changed at the level of 0.0002 Å. This shows, that restraint assignments indeed change over the course of the refinement, and that in practical applications the restraints should to be updated whenever the model geometry changes, or pragmatically—before each refinement cycle.

**Figure 5.**
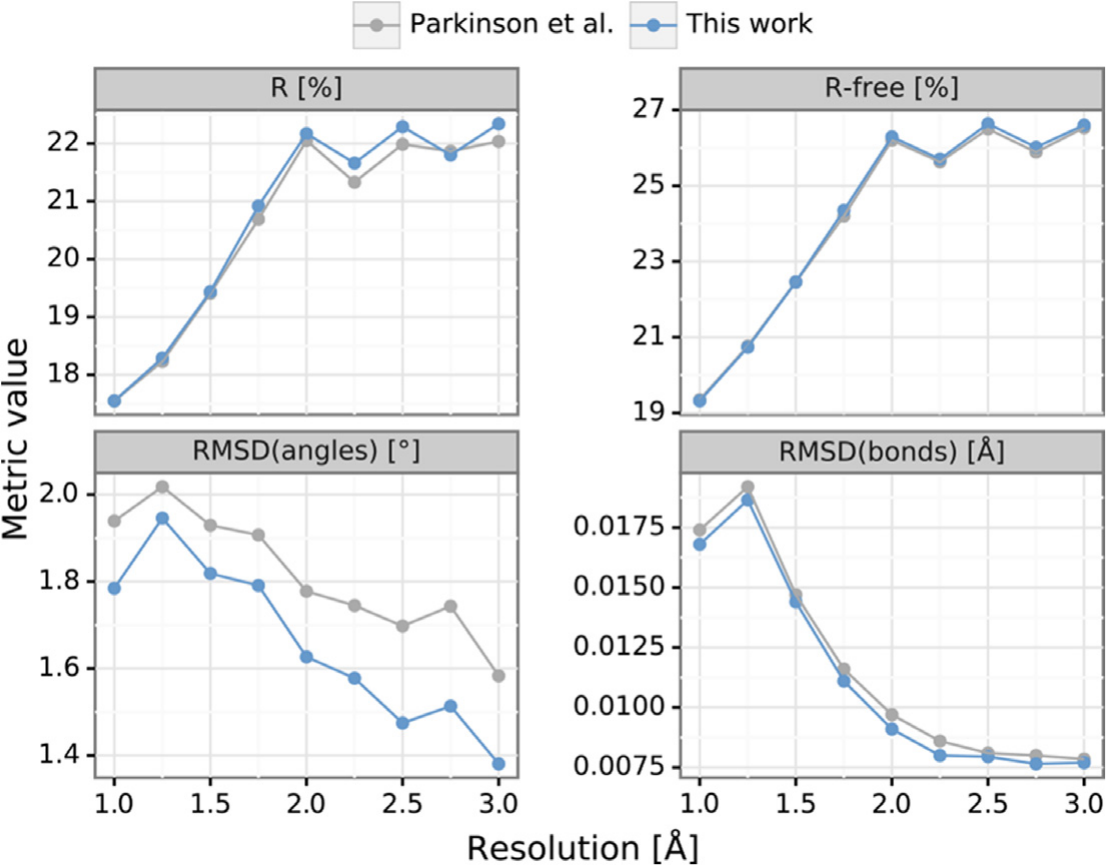
Comparison of median *R*, *R*_free_, RMSD(bonds) and RMSD(angles) of 617 nucleic acid structures from the PDB with resolution between 1.0 and 3.0 Å. The structures were re-refined using five cycles of REFMAC with default Parkinson restraints (gray) and with the sugar restraints proposed in this work (blue).

Finally, in an attempt to verify the robustness of the proposed sugar restraints on a larger set of structures, we generated sugar restraints for 1565 PDB deposits covering all X-ray nucleic acid structures without proteins and with resolution between 1.0 and 3.0 Å. To perform such a massive experiment, we added a REST API functionality to the RestraintLib server, which allows programmatic retrieval of restraints without having to visit the website. Out of the 1565 structures, we selected a subset of 617 deposits (Supplementary Table S4) that had at least 400 *R*_free_ reflections, had <50% residues with alternative conformations, and had experimental diffraction data that could be successfully converted to MTZ format (35). Each of those structures was refined by five cycles of REFMAC (30,31), first using default restraints and then using the restraints proposed in this work. The results were compared by means of *R*, *R*_free_, RMSD(bonds) and RMSD(angles). According to a series of Wilcoxon signed-rank tests with Bonferroni correction, when using the proposed restraints *R* slightly increased (*p* < 0.001), *R*_free_ did not change (*p* = 0.238), whereas both RMSD(bonds) and RMSD(angles) were significantly improved (*p* < 0.001). The RMSD(bonds) and RMSD(angles) improved for 75% and 92% of the cases, respectively. The median improvement with the use of RestraintLib was 0.0004 Å for RMSD(bonds) and 0.11° for RMSD(angles), with *R*/*R*_free_ staying practically unchanged (–0.08%/0.00%). Taking into account that those structures are of average quality and had been mostly refined using Parkinson restraints, the RMSD improvement can be viewed as substantial. Moreover, an analysis of the above-mentioned metrics for different resolution intervals (Figure 5) shows that the improvements are consistent for all resolution ranges. The medians of *R* and *R*_free_ are practically identical when either the default or the newly proposed restraints are used, regardless of resolution. RMSD(bonds) and RMSD(angles) are consistently better when the proposed restraints are used, with the improvement of RMSD(angles) being higher (∼0.20°) at lower resolution (*d*_min_ ≥ 2 Å).

### Updated RestraintLib server

The restraints for the sugar moieties of nucleic acids described in this paper can be generated automatically using our RestraintLib server (http://achesym.ibch.poznan.pl/restraintlib/). Upon input of a suitable PDB or mmCIF file, the server produces a file with all the bond length and bond angle restraints in REFMAC (30,31), PHENIX (32) or SHELXL (33) format, with sigmas taken directly from the standard deviations reported in Tables 1–3. RestraintLib can now be also integrated with existing refinement software through a programmatic API. At present, the server is capable of generating conformation-dependent restraints for the entire nucleic acid structure, including the phosphodiester (11), nucleobase (12), and sugar (this work) moieties. The server is also capable of producing restraints for alternative conformations.

## CONCLUSIONS

In this paper, we have described a new set of restraints for the sugar moiety of nucleic acids. The proposed restraints are conformationally-dependent within groups or as functional relationships. Functional relationships found for endocyclic ribose angles, as well as for bonds and angles depending on the glycosidic torsion angle χ, could prove very useful in determining the geometric parameters of structures with rare or otherwise unusual conformations, previously not seen even in the CSD. When validated against NDB data and an ultrahigh-resolution structure from the PDB, the new restraints show significantly better agreement with experimental data than the standard nucleic acid restraint library of Parkinson *et al.* (3). Moreover, our conformation-dependent sugar restraints can be easily generated for any standard PDB or mmCIF file using an updated version of our RestraintLib web server, which also includes a REST API functionality for programmatic access.

With this paper we have completed the revision of stereochemical restraints for all three building blocks of the nucleotide unit in nucleic acids structures. We have seen that the bases are relatively rigid fragments, whereas the phosphate group and sugar moiety, forming the nucleic acid backbone, are clearly more flexible, and can assume a large number of conformational states, either discrete or continuous. In the future, it might be possible to find out that more parameters are functionally dependent on conformation, yet this would require more data. It is also worth noting that many of the nucleoside/nucleotide structures found in the CSD are relatively old. Reinvestigation of those structures using currently available state-of-the-art equipment and methodology could significantly improve studies like this.

Finally, the applications of machine learning and automation procedures in this project have led us to the conclusion that future updates of restraint libraries could be carried out automatically as the volume of structural data expands. Work on an automated version of RestraintLib is in progress.

## DATA AVAILABILITY

Source code and reproducible experimental scripts for this paper are available on GitHub at https://github.com/dabrze/sugar_conformation.

## Supplementary Material

gkz1122_Supplemental_File

## References

[1] Berman H.M., Westbrook J., Feng Z., Gilliland G., Bhat T.N., Weissig H., Shindyalov I.N., Bourne P.E. The Protein Data Bank. Nucleic Acids Res. 2000; 28:235–242. 10592235 10.1093/nar/28.1.235 PMC102472

[2] Taylor R., Kennard O. The molecular structures of nucleosides and nucleotides. Part 1. The influence of protonation on the geometries of nucleic acid constituents. J. Mol. Struct. 1982; 78:1–28.

[3] Parkinson G., Vojtechovsky J., Clowney L., Brünger A.T., Berman H.M. New parameters for the refinement of nucleic acid-containing structures. Acta Crystallogr. 1996; D52:57–64. 10.1107/S0907444995011115 15299726

[4] Gelbin A., Schneider B., Clowney L., Hsieh S., Olson W.K., Berman H.M. Geometric parameters in nucleic acids: sugar and phosphate constituents. J. Am. Chem. Soc. 1996; 118:519–529.

[5] Clowney L., Jain S.C., Srinivasan A.R., Westbrook J., Olson W.K., Berman H.M. Geometric parameters in nucleic acids: nitrogenous bases. J. Am. Chem. Soc. 1996; 118:509–518.

[6] Groom C.R., Bruno I.J., Lightfoot M.P., Ward S.C. The Cambridge Structural Database. Acta Crystallogr. 2016; B72:171–179. 10.1107/S2052520616003954 PMC4822653 27048719

[7] Tronrud D.E., Karplus P.A. A conformation-dependent stereochemical library improves crystallographic refinement even at atomic resolution. Acta Crystallogr. 2011; D67:699–706. 10.1107/S090744491102292X PMC3144852 21795811

[8] Moriarty N.W., Tronrud D.E., Adams P.D., Karplus P.A. Conformation-dependent backbone geometry restraints set a new standard for protein crystallographic refinement. FEBS J. 2014; 281:4061–4071. 24890778 10.1111/febs.12860 PMC4169323

[9] Moriarty N.W., Tronrud D.E., Adams P.D., Karplus P.A. A new default restraint library for the protein backbone in Phenix: a conformation-dependent geometry goes mainstream. Acta Crystallogr. 2015; D72:176–179. 10.1107/S2059798315022408 PMC4756615 26894545

[10] Jaskolski M., Gilski M., Dauter Z., Wlodawer A. Stereochemical restraints revisited: how accurate are refinement targets and how much should protein structures be allowed to deviate from them. Acta Crystallogr. 2007; D63:611–620. 10.1107/S090744490700978X 17452786

[11] Kowiel M., Brzezinski D., Jaskolski M. Conformation-dependent restraints for polynucleotides: I. Clustering of the geometry of the phosphodiester group. Nucleic Acids Res. 2016; 44:8479–8489. 27521371 10.1093/nar/gkw717 PMC5041494

[12] Gilski M., Zhao J., Kowiel M., Brzezinski D., Turner D.H., Jaskolski M. Accurate geometrical restraints for Watson–Crick base pairs. Acta Crystallogr. 2019; B75:235–245. 10.1107/S2052520619002002 PMC6457083 32830749

[13] Altona C., Sundarlingam M. Conformational analysis of the sugar ring in nucleosides and nucleotides. New description using the concept of pseudorotation. J. Am. Chem. Soc. 1972; 94:8205–8212. 5079964 10.1021/ja00778a043

[14] De Leeuw H.P.M., Haasnoot C.A.G., Altona C. Empirical correlations between conformational parameters in β-D-furanoside fragments derived from a statistical survey of crystal structures of nucleic acid constituents. Full description of nucleoside molecular geometries in terms of four parameters. Israel J. Chem. 1980; 20:108–126.

[15] Bruno I.J., Cole J.C., Edgington P.R., Kessler M., Macrae C.F., McCabe P., Pearson J., Taylor R. The Cambridge Structural Database. Acta Crystallogr. 2002; B58:389–397. 10.1107/s0108768102003324 12037360

[16] Iglewicz B., Hoaglin D. How to Detect and Handle Outliers. 1993; Milwaukee ASQC Quality Press.

[17] Jaskolski M. A comparison of two methods for the calculation of pseudorotation parameters. Acta Crystallogr. 1984; A40:364–366.

[18] Welch B.L. The generalization of “Student's” problem when several different population variances are involved. Biometrika. 1947; 34:28–35. 20287819 10.1093/biomet/34.1-2.28

[19] Myers J.L., Well A.D. Research Design and Statistical Analysis (2nd edn). 2003; Lawrence Erlbaum.

[20] Rasmussen C.E., Williams C.K.I. Gaussian Processes for Machine Learning. 2006; MIT Press.

[21] Oliphant T.E. Python for scientific computing. Comput. Sci. Eng. 2007; 9:10–20.

[22] Pedregosa F., Varoquaux G., Gramfort A., Michel V., Thirion B., Grisel O., Blondel M., Prettenhofer P., Weiss R., Dubourg V. et al. Scikit-learn: machine learning in Python. J. Mach. Learn. Res. 2011; 12:2825–2830.

[23] Shapiro S.S., Wilk M.B. An analysis of variance test for normality (complete samples). Biometrika. 1965; 52:591–611.

[24] Narayanan B.C., Westbrook J., Ghosh S., Petrov A.I., Sweeney B., Zirbel C.L., Leontis N.B., Berman H.M. The Nucleic Acid Database: new features and capabilities. Nucleic Acids Res. 2014; 42:D114–D122. 24185695 10.1093/nar/gkt980 PMC3964972

[25] Brzezinski K., Brzuszkiewicz A., Dauter M., Kubicki M., Jaskolski M., Dauter Z. High regularity of Z-DNA revealed by ultra high-resolution crystal structure at 0.55 Å. Nucleic Acids Res. 2011; 39:6238–6248. 21459852 10.1093/nar/gkr202 PMC3152349

[26] Demšar J. Statistical comparisons of classifiers over multiple data sets. J. Mach. Learn. Res. 2006; 7:1–30.

[27] Jakob M., Kolodziejczyk R., Orlowski M., Krzywda S., Kowalska A., Dutko-Gwozdz J., Gwozdz T., Kochman M., Jaskolski M., Ozyhar A. Novel DNA-binding element within the C-terminal extension of the nuclear receptor DNA-binding domain. Nucleic Acids Res. 2007; 35:2705–2718. 17426125 10.1093/nar/gkm162 PMC1885670

[28] Wedekind J.E., McKay D.B. Crystal structure of a lead-dependent ribozyme revealing metal binding sites relevant to catalysis. Nat. Struct. Mol. Biol. 1999; 6:261–268. 10.1038/6700 10074945

[29] Drozdzal P., Gilski M., Kierzek R., Lomozik L., Jaskolski M. High-resolution crystal structure of Z-DNA in complex with Cr(3+) cations. J. Biol. Inorg. Chem. 2015; 20:595–602. 25687556 10.1007/s00775-015-1247-5 PMC4381091

[30] Murshudov G.N., Skubák P., Lebedev A.A., Pannu N.S., Steiner R.A., Nicholls R.A., Winn M.D., Long F., Vagin A.A. REFMAC5 for the refinement of macromolecular crystal structures. Acta Crystallogr. 2011; D67:355–367. 10.1107/S0907444911001314 PMC3069751 21460454

[31] Kovalevskiy O., Nicholls R.A., Long F., Murshudov G.N. Overview of refinement procedures within REFMAC5: utilizing data from different sources. Acta Crystallogr. 2018; D74:492–505. 10.1107/S2059798318000979 PMC5947762 29533229

[32] Adams P.D., Afonine P.V., Bunkóczi G., Chen V.B., Davis I.W., Echols N., Headd J.J., Hung L.-W., Kapral G.J., Grosse-Kunstleve R.W. et al. PHENIX: a comprehensive Python-based system for macromolecular structure solution. Acta Crystallogr. 2010; D66:213–221. 10.1107/S0907444909052925 PMC2815670 20124702

[33] Sheldrick G.M. Crystal structure refinement with SHELXL. Acta Crystallogr. 2015; C71:3–8. 10.1107/S2053229614024218 PMC4294323 25567568

[34] Nicholls R.A., Long F., Murshudov G.N. Low-resolution refinement tools in REFMAC5. Acta Crystallogr. 2012; D68:404–417. 10.1107/S090744491105606X PMC3322599 22505260

[35] Winn M.D., Ballard C.C., Cowtan K.D., Dodson E.J., Emsley P., Evans P.R., Keegan R.M., Krissinel E.B., Leslie A.G., McCoy A. et al. Overview of the CCP4 suite and current developments. Acta Crystallogr. 2011; D67:235–242. 10.1107/S0907444910045749 PMC3069738 21460441

