# Supplementary material for: Conformation-dependent restraints for polynucleotides: the sugar moiety
Source: Nucleic Acids Res. 2019 Dec 4;48(2):962–73. doi: 10.1093/nar/gkz1122 (PMC6954431; doi:10.1093/nar/gkz1122)
Supplement: gkz1122_Supplemental_File [file gkz1122_supplemental_file.pdf]

Conformation-dependent restraints for polynucleotides: The sugar moiety. Supplementary materials.

Marcin Kowiel[1], Dariusz Brzezinski[1,2,3], Miroslaw Gilski[1,4] and Mariusz Jaskolski[1,4*]

[1]Center for Biocrystallographic Research, Institute of Bioorganic Chemistry, Polish Academy of Sciences, Poznan, 61-714, Poland, [2]Institute of Computing Science, Poznan University of Technology, Poznan, 60-965, Poland, [3]Center for Artificial Intelligence and Machine Learning, Poznan University of Technology, Poznan, 60-965, Poland, [4]Department of Crystallography, Faculty of Chemistry, A. Mickiewicz University, Poznan, 61-614, Poland;

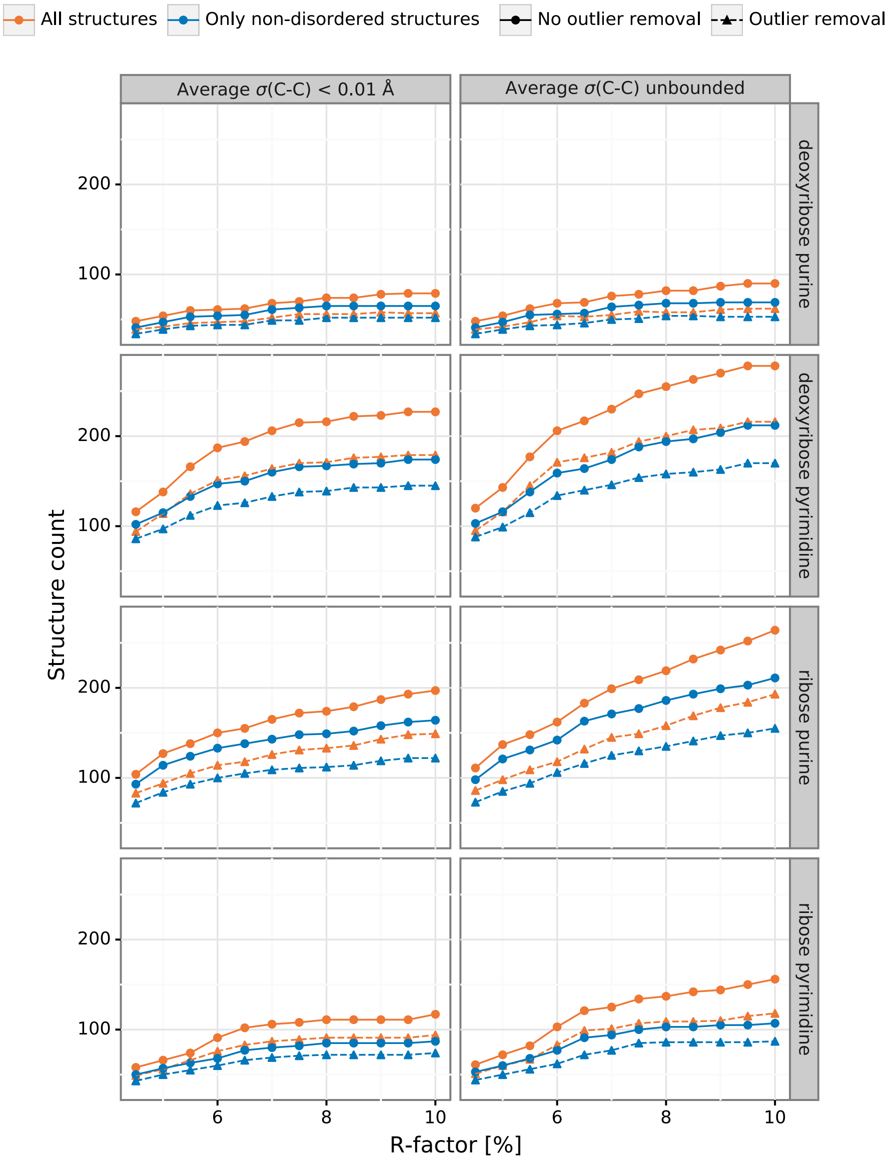

Supplementary Figure S1. Number of sugar substructures in the CSD retrieved using various sampling criteria: *R*-factor threshold (x-axis), maximum σ(C-C) (columns), all/non-disordered structures (blue/orange), and all structures/outlier removal (solid/dash line).

*Tel: +48-61-829-1274; Fax: +48-61-829-1555; Email: mariuszj@amu.edu.pl

© 2016 Marcin Kowiel, Dariusz Brzezinski, and Mariusz Jaskolski
This is an Open Access article distributed under the terms of the Creative Commons Attribution Non-Commercial License (http://creativecommons.org/licenses/by-nc/2.0/uk/) which
permits unrestricted non-commercial use, distribution, and reproduction in any medium, provided the original work is properly cited.

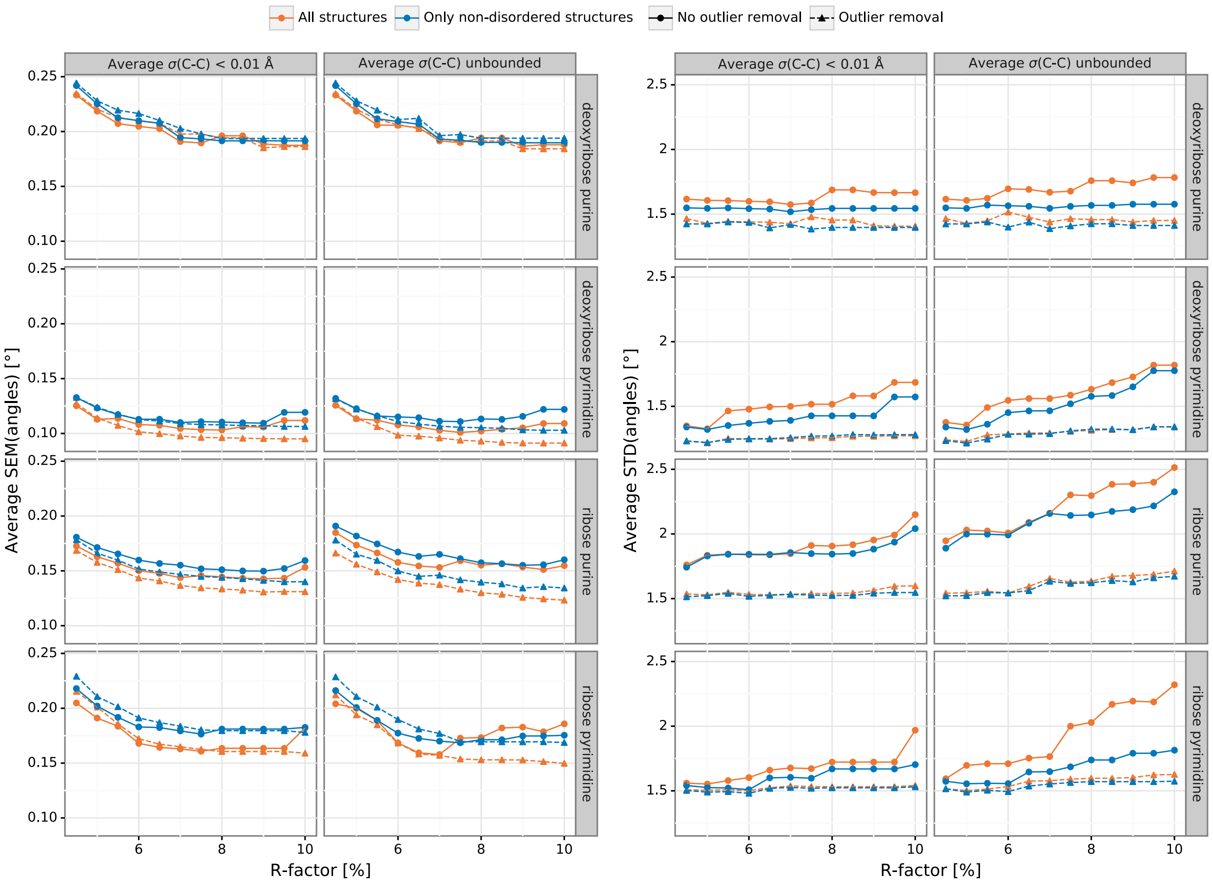

Supplementary Figure S2. Analysis of average standard error of the mean for bond angles (SEM(angles), left) and average sample standard deviation (STD(angles), right) for varying CSD sampling criteria: *R*-factor threshold (x-axis), maximum σ(C-C) (columns), all/non-disordered structures (blue/orange), and all structures/outlier removal (solid/dash line).

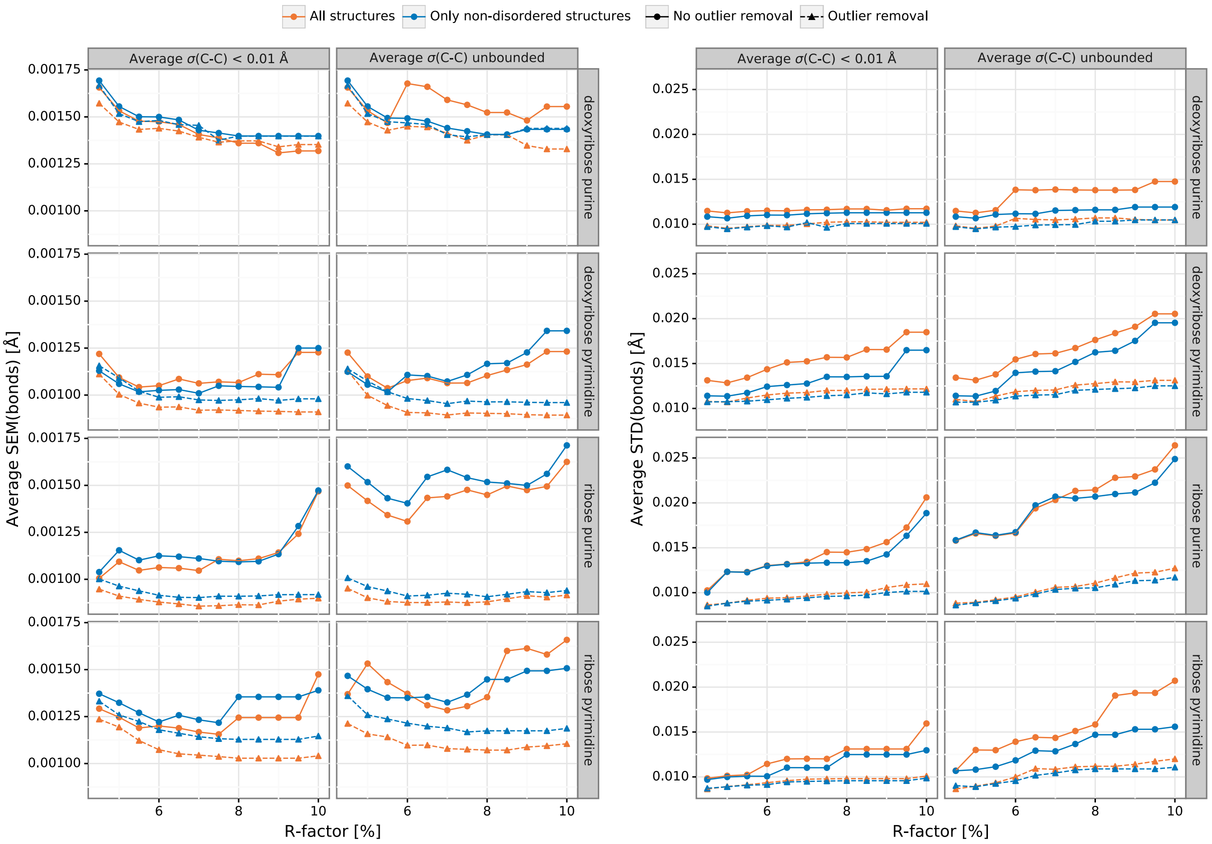

Supplementary Figure S3. Analysis of average standard error of the mean for bond lengths (SEM(bonds)) (left) and average sample standard deviation (STD(bonds)) (right) for varying CSD sampling criteria: *R*-factor threshold (x-axis), maximum σ(C-C) (columns), all/non-disordered structures (blue/orange), and all structures/outlier removal (solid/dash line).

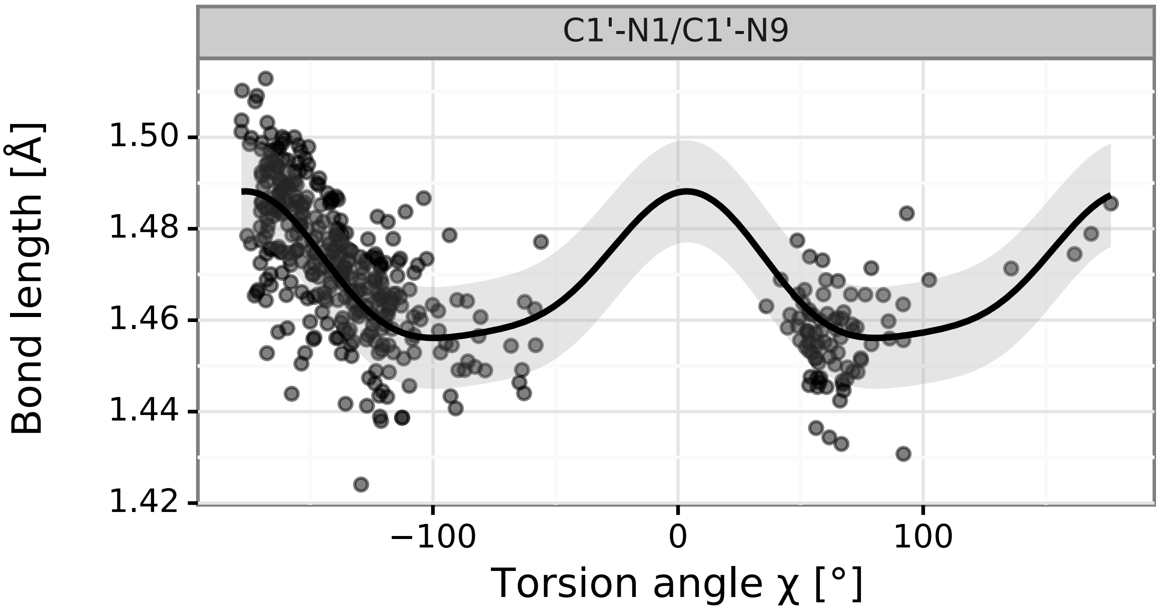

Supplementary Figure S4. Scatterplot presenting the mean (line) and standard error (semi-transparent band) calculated by Gaussian Process Regression for the glycosidic bond C1’-N1/C1’-N9.

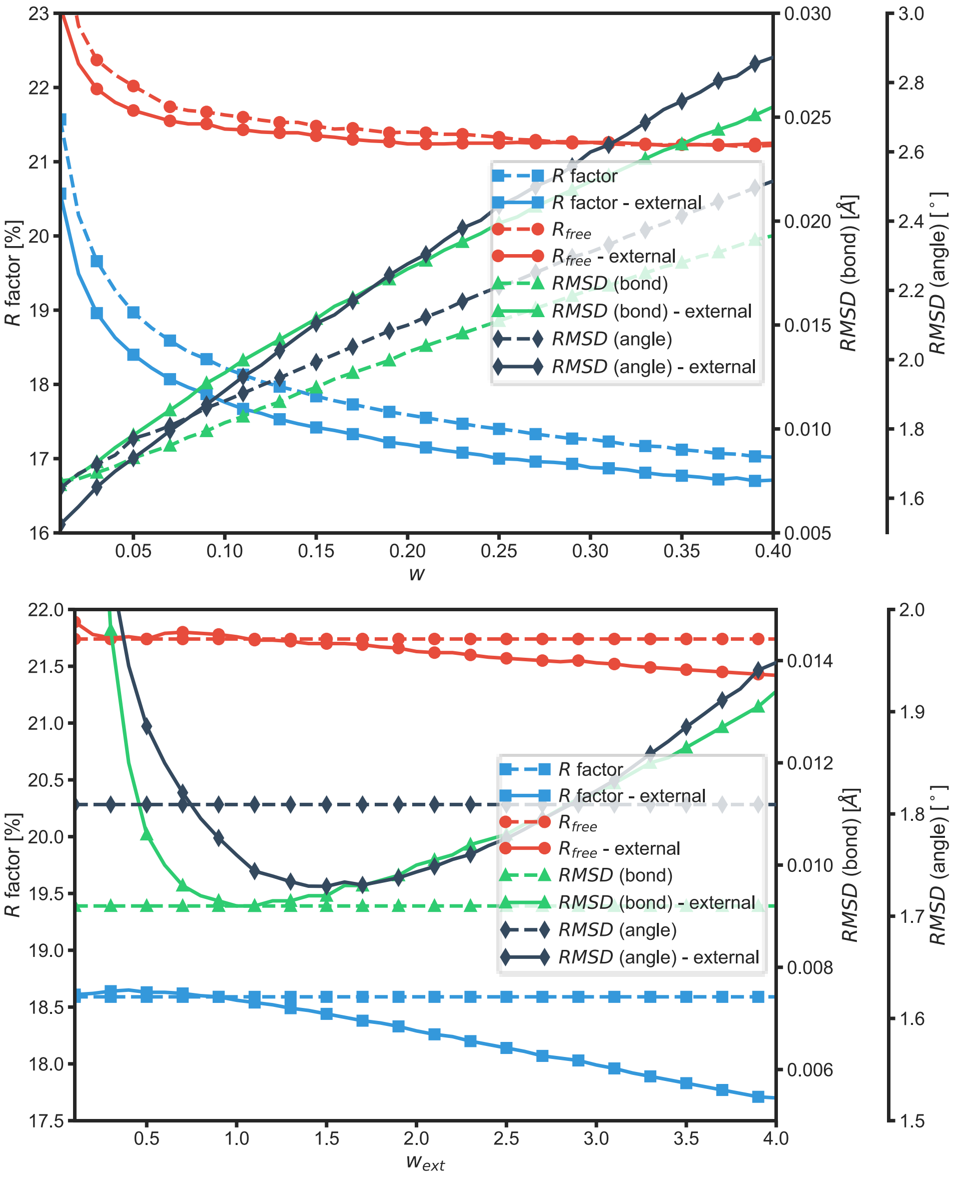

Supplementary Figure S5. 2HAN Refinement parameters. *R*-factor (blue lines, left y-axis), R_{free} (red lines, left y-axis) and RMSD(bonds) (green lines, right y-axis), RMSD(angles) (black lines, right y-axis) after 30 REFMAC iterations for the PDB 2HAN model without (dash lines) and with (solid lines) external sugar restraints, plotted against: (top) the weight parameter w (w_{ext} = 2.7, κ = 0.5); (bottom) external weight scale w_{ext} (w = 0.07, κ = 0.5).

The statistics for optimal parameter values are presented in Table 4 of the main manuscript. First, we found the value of w (0.07) that gives an RMSD(angles) of ~1.80° (green dash line in top panel). Since w is the only parameter that is common to both refinements, fixing its value makes the refinements with and without external restraints comparable. Next, we attempted to find such a value of w_{ext} that would give the best values of R and R_{free}, while keeping RMSD(angles) at 1.80° (solid green line in bottom panel). In the analyzed example, changing κ between 0.5 and 5 had no effect on model statistics. The presented plots show that the new sugar restraints can be used to obtain better RMSD values for the same R/R_{free} statistics, or to obtain improved R/R_{free} factor statistics at the same level of RMSD(angles).

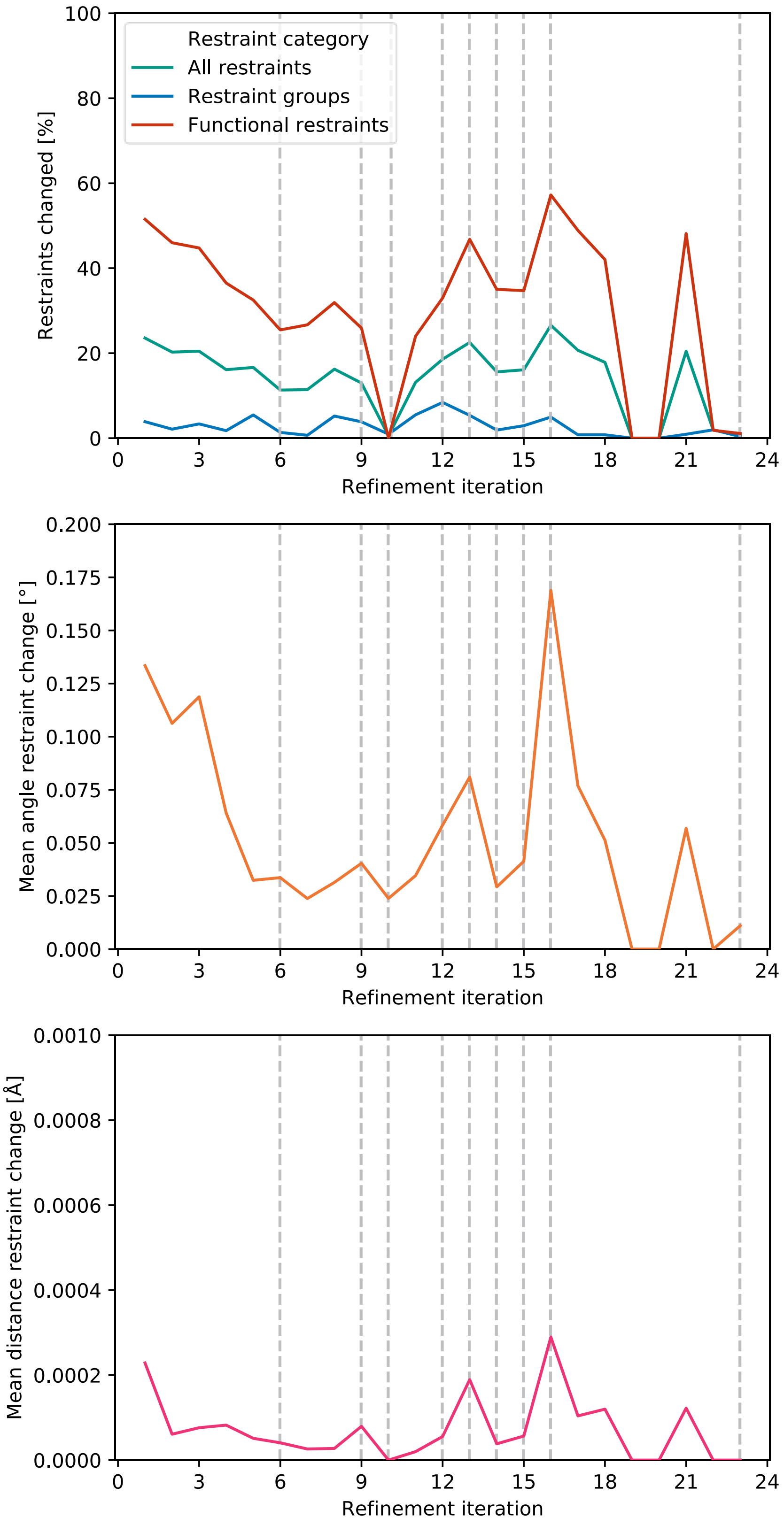

Supplementary Figure S6. Analysis of sugar restraint changes during the refinement of a novel 1.6 Å RNA structure: (top) percentage of restraints that have changed between a pair of refinement iterations, (middle) mean change per angle restraint (in °), (bottom) mean change per distance restraint (in Å). Dash lines mark iterations where alternative conformations were added to the model.

Table S1. CSD refcodes of structures used to determine the geometry of the indicated sugars.

**ribose-purine (R-Pu)**					
ABELUP	ACADOS	ADENOS01	ADENOS10	ADENOS13	ADMOPM
ADMPOT10	ADOSBU01	ADOSHC	ADPOSD	ADYPUR10	AFURBA
AMADPH10	APAPAD10	ARADEN10	ARFMAD	ARFUAD	ARFUAD01
BARFUP	BERNOB	BIMFEI	BIMFIM	BRADOS01	BRGUOS01
BRINOS10	CAINSP	CAKNIO	CAKNOU	CATGIP	CATGOV
CATGUB	CEVYEH01	CEVYEH11	CIMTEX	CLPURB	COCZID
COXNEI	CRFIMZ	CUTVAO	DAXTOO	DAZADN10	DEHQOW
DEMXEY	DMGUAN10	DOHSIC	DUVHEH10	EADNPP	ECAFEU
ECEGOJ	ERFIMP	FASDUZ	FEYBER	FUCWIJ	GEXGAR
GEXGAR01	GMPCUP	GODPIY	GOHSAZ	GUANSH10	GUOPNA10
GUOPNA11	GUPCYT20	HECCAT	HEXBAO	HIFKEM	HIPADS
HIQNAW	INOSIN10	INOSIN11	INOSND10	INPOSR	JAZZOZ10
JEMHOZ	JIGCUX	KIKPUP	KIPFUN	LEBLOW	MEQLUS
MGUOSM	MGUOSM01	MOPRBS	MRFPUR	MSPURI	MSPURI01
NAQFOC	NAQFUI	NAXHOL	NBTINS10	NEBULR	NEBULR01
NIVTET	PCTRIB10	PUCGLR10	PUPZAC	QAGFAH	ROBZIR
SELZIS	SITMIR	SITVIA	TGUANS10	THPRIB	TIBWOQ
TUVZIU	WEKKON	WEKKUT	XANTOS	XUQJEZ	XUXYEV
XYFUAD10	YANDOJ	ZADENH10	ZAYTOI	ZEXWEE	
**deoxyribose-purine (D-Pu)**					
ASADED	BAGVEK	CEZMEZ	DADPNH10	DEFPOT	DEHREN
DEHREN01	DINYII10	DTGUOS	EBOYAY	EHEDOK	FIKHAI
FOYLUA	FUXKAL	FUXKEP	GUZPOH	IFOSAY	IKOMAY
JAFHIH	KEBWOF	KEBWUL	KECLOT	KOKPOP	MOFBUE
NALCUB	NEJXOQ	NEJXUW	PITVUK	PITVUK01	PITWEV
PITWIZ	PITWOF	QIKFAU	RIQMEK	RITNAK	SDGUNP
TELXOA	UYOCAO	VICGET	XIZGIW	XIZGOC	XUQJID
XUQJOJ	XUQJUP	XUQKAW	ZOZXOB	ZUWNUA	
**ribose-pyrimidine (R-Py)**					
ACYTID	ADYPUR10	ARBCYT10	ARCMPH01	ARFCYT10	BAGXIQ01
BEURID10	BEZGES	BICVEO	CADZAI	CBMURD	CEFJUS
CEKLUZ	CERYED	CUNSAF	CUPYUH	CXMURD	CYTIAC
CYTIAC01	CYTICL	CYTIDI01	CYTIDI02	CYTIDI10	CYTIDI11
CYTIDI12	CYTIDN	DAURID01	DIPCOU10	EJATOA	FOCFOT
GICMOU	GODPOE	GUPCYT20	GUTPUI	GUTQAP	GUTQET
HIKCAH	HIKNIY	HIMXIL	JUCJAS	KECCUT	KIGLUJ
KIGMAQ	KIGMEU	KIGMOE	LIGXIL	LOWJIQ	LYFURA
MARAFC	MEURID	MEURID02	MEURID03	MUCWOY	PUCPAF
ROVQEA	SALCYS	SATCEW	SURIDP	SUROMM	TAHXOS
TALJAR	TONQIX	URARAF01	URARAF10	URIDMP10	VIKKIK
VIKKIK01	VUYMAD	XANBEU	XFURCC10	XOMKOB	XOMKUH
XYFCYT10	YIDKIF	ZAYTIC	ZOYLEE		
**deoxyribose-pyrimidine (D-Py)**					
ACDXUR	ADUPIY	AKUZEM	BEBXEL	BIXNIG	BIXNOM
BOXGIE	CABSIH10	CASKAI	CECYIU	CITMOH01	COKBIP
CUKHEW	DEJSAM	DINYII10	DOCYPO	DOCYPO03	DOCYPO04
DOCYTC	DONBUD	DOURID	DOXPOV	DUHHUJ	DXCYTD
DXCYTD01	EHUFEU	ETYXUR	EWALAR	EYOJUY	FEFMEK
FESCIQ	FEVDEQ	FIHXID	FIHXUP	FIQSEF	FMDURD02

FUWHEL	FUXBIJ	FUXBIJ01	GEBTEM	GERGAL	GEXXIQ
GEXXIQ01	GIDTIW	GODTOI	GOQCAS	HAXRAB	HECMAG
HEVXOV	HEZWAL	HEZWEP	HEZWIT	HEZWOZ	HIFVEY
HOLSUY	IJIHIU	IJIHOA	IJIHUG	IPDXUR	IPDXUR01
JAJQAM	JAPPEV	JIBDIH	KAMCUW	KASVEF	KECRUI
KECSAP	LIJZIN	LOGYUC	LOWHEK	LOWHIO	LOWHOU
LOWJEM	MOLQUZ	MOLRAG	NEKBOV	NULJAG	NULJIO
PAVYOB	POGGAT	PULVIB	QAJJES	QOKJUV	RAKLOG
REJHOG	REJJAU	REMSOR	RESMAD	RIDGOC	ROHHII
ROHHOO	SEDQEX	SERQUC	SERRAJ	SIFGAP	SIFKEX
SOLLAH	SONDOO	SULZEF	THYDIN	THYDIN01	THYDIN02
THYDIN03	THYDIN04	THYDIN05	THYDIN06	THYMDN01	TUFFUV
TUFGEG	UFEWEJ	VAWNEO	VEXDOR	WAZZAA	WEVJOX
WEVWIE	WIKPEL	WISGEK	XAWYIF	XEWQUL	XEZCAH
XIMMIQ	YAGSAC	YEGSEI	YEVJAK	YEWCEJ	YUYJUX
ZAPWOC	ZASQUF	ZASRAM	ZATCOP	ZATCUV	ZOZVUF

Table S2. CSD refcodes of structures used to determine the geometry of terminal sugars.

**terminal C3' ribose-purine (R-Pu)**					
ABELUP	ADENOS01	ADENOS10	ADENOS13	ADMOPM	ADMPOT10
ADOSBU01	ADOSHC	AFURBA	ARADEN10	ARFMAD	ARFUA
ARFUAD01	BARFUP	BERNOB	BIMFEI	BIMFIM	BRADOS01
BRGUOS01	BRINOS10	CAINSP	CAKNIO	CAKNOU	CATGIP
CATGOV	CATGUB	CEVYEH01	CEVYEH11	CIMTEX	CLPURB
COCZID	COXNEI	CRFIMZ	CUTVAO	DAZADN10	DEHQOW
DEMXEY	DMGUAN10	DOHSIC	DUVHEH10	EADNPP	ECAFEU
ECEGOJ	ERFIMP	FASDUZ	FEYBER	FUCWIJ	GEXGAR
GEXGAR01	GODPIY	GOHSAZ	GUANSH10	GUOPNA10	GUOPNA11
HECCAT	HEXBAO	HIFKEM	HIPADS	HIQNAW	INOSIN10
INOSIN11	INOSND10	INPOSR	JAZZOZ10	JEMHOZ	JIGCUX
KASZUZ	KIKPUP	LEBLOW	MEQLUS	MGUOSM	MGUOSM01
MOPRBS	MRFPUR	MSPURI	MSPURI01	NAQFOC	NAQFUI
NAXHOL	NBTINS10	NEBULR	NEBULR01	NIVTET	PCTRIB10
PUCGLR10	PUPZAC	QAGFAH	ROBZIR	SELZIS	TGUANS10
THPRIB	TIBWOQ	TUVZIU	WEKKON	WEKKUT	XANTOS
XUQJEZ	XUXYEV	XYFUAD10	YANDOJ	ZADENH10	ZAYTOI
ZEXWEE					
**terminal C3' deoxyribose-purine (D-Pu)**					
ASADED	BAGVEK	CEZMEZ	DADPNH10	DEFPOT	DEHREN
DEHREN01	DINYII10	DTGUOS	EBOYAY	EHEDOK	FUXKAL
FUXKEP	IFOSAY	IKOMAY	KECLOT	KOKPOP	NEJXOQ
PITVUK	PITVUK01	PITWEV	PITWIZ	PITWOF	RITNAK
SDGUNP	TELXOA	UYOCAO	VICGET	XUQJID	XUQJOJ
XUQJUP	XUQKAW	ZOZXOB	ZUWNUA		
**terminal C3' ribose-pyrimidine (R-Py)**					
ACYTID	ADYPUR10	ARBCYT10	ARCMPH01	ARFCYT10	BEURID10
BEZGES	CADZAI	CBMURD	CEFJUS	CEKLUZ	CERYED
CUNSAF	CUPYUH	CXMURD	CYTICL	CYTIDI01	CYTIDI02
CYTIDI10	CYTIDI11	CYTIDI12	CYTIDN	DIPCOU10	EJATOA
FOCFOT	GICMOU	GODPOE	GUPCYT20	GUTPUI	GUTQAP
GUTQET	HIKCAH	HIKNIY	HIMXIL	JUCJAS	KECCUT
KIGLUJ	KIGMAQ	KIGMEU	KIGMOE	LIGXIL	LOWJIQ
LYFURA	MEURID	MEURID02	MEURID03	MUCWOY	ROVQEA
SALCYS	SATCEW	SUROMM	TAHXOS	TALJAR	TONQIX
URARAF01	URARAF10	VIKKIK	VIKKIK01	VUYMAD	XFURCC10
XOMKOB	XOMKUH	XYFCYT10	YIDKIF	ZAYTIC	
**terminal C3' deoxyribose-pyrimidine (D-Py)**					
ACDXUR	BEBXEL	BIXNIG	BIXNOM	CABSIH10	CASKAI
CECYIU	CITMOH01	CUKHEW	DOCYPO	DOCYPO03	DOCYPO04
DOCYTC	DONBUD	DOURID	DOXPOV	DUHHUJ	DXCYTD
DXCYTD01	ETYXUR	EWALAR	FEFMEK	FESCIQ	FIHXID
FIQSEF	FMDURD02	FUWHEL	GEBTEM	GERGAL	GIDTIW
GODTOI	GOQCAS	HAXRAB	HECMAG	HEVXOV	HEZWAL
HEZWEP	HEZWIT	HEZWOZ	HIFVEY	HOLSUY	IJIHIU
IJIHOA	IJIHUG	IPDXUR	IPDXUR01	JAPPEV	JIBDIH
KAMCUW	KECRUI	KECSAP	LIJZIN	LOGYUC	LOWHEK
LOWHIO	LOWHOU	LOWJEM	MOLQUZ	MOLRAG	NEKBOV
POGGAT	PULVIB	RAKLOG	REMSOR	RESMAD	RIDGOC

ROHHII	ROHHOO	SEDQEX	SERQUC	SERRAJ	SIFKEX
SOLLAH	SULZEF	THYDIN	THYDIN01	THYDIN02	THYDIN03
THYDIN04	THYDIN05	THYDIN06	THYMDN01	TUFFUV	TUFGEG
UFEWEJ	VAWNEO	VEXDOR	WAZZAA	WEVWIE	WIKPEL
WISGEK	XAWYIF	YEWCEJ	ZAPWOC	ZOZVUF	
**terminal C5' ribose-purine (R-Pu)**					
ABELUP	ACADOS	ADENOS01	ADENOS10	ADENOS13	ADOSBU01
ADOSHC	ADPOSD	ADYPUR10	ARADEN10	ARFUAD	ARFUAD01
BARFUP	BERNOB	BIMFEI	BIMFIM	BRADOS01	BRGUOS01
BRINOS10	CAKNIO	CAKNOU	CATGIP	CATGOV	CATGUB
CIMTEX	CLPURB	COXNEI	CRFIMZ	CUTVAO	DAZADN10
DEHQOW	DEMXEY	DMGUAN10	DOHSIC	ECAFEU	ECEGOJ
ERFIMP	FUCWIJ	GEXGAR	GEXGAR01	GUANSH10	GUPCYT20
HECCAT	HEXBAO	HIPADS	HIQNAW	INOSIN10	INOSIN11
INOSND10	JAZZOZ10	JIGCUX	KASZUZ	LEBLOW	MGUOSM
MGUOSM01	MOPRBS	MRFPUR	MSPURI	MSPURI01	NAQFOC
NAQFUI	NBTINS10	NEBULR	NEBULR01	PCTRIB10	PUCGLR10
PUPZAC	ROBZIR	SELZIS	SITMIR	SITVIA	TGUANS10
THPRIB	TIBWOQ	WEKKON	WEKKUT	XANTOS	XUQJEZ
XUXYEV	XYFUAD10	YANDOJ	ZAYTOI	ZEXWEE	
**terminal C5' deoxyribose-purine (D-Pu)**					
ASADED	BAGVEK	DEFPOT	DEHREN	DEHREN01	DTGUOS
EBOYAY	EHEDOK	FOYLUA	FUXKAL	FUXKEP	IFOSAY
IKOMAY	KECLOT	KOKPOP	NEJXOQ	PITVUK	PITVUK01
PITWEV	PITWIZ	PITWOF	RIQMEK	RITNAK	TELXOA
UYOCAO	VICGET	XUQJID	XUQJOJ	XUQJUP	XUQKAW
ZOZXOB	ZUWNUA				
**terminal C5' ribose-pyrimidine (R-Py)**					
ACYTID	ARBCYT10	ARFCYT10	BEURID10	BEZGES	CADZAI
CBMURD	CEFJUS	CERYED	CUNSAF	CXMURD	CYTIAC
CYTIAC01	CYTICL	CYTIDI01	CYTIDI02	CYTIDI10	CYTIDI11
CYTIDI12	CYTIDN	DIPCOU10	EJATOA	ETCYTC	GICMOU
GODPOE	HIKCAH	HIKNIY	JUCJAS	LYFURA	MARAFC
MCYTMS10	MEURID	MEURID02	MEURID03	SALCYS	SURIDP
TAHXOS	TALJAR	TONQIX	URARAF01	URARAF10	VIKKIK
VIKKIK01	VUYMAD	XOMKOB	YIDKIF	ZAYTIC	
**terminal C5' deoxyribose-pyrimidine (D-Py)**					
ACDXUR	BEBXEL	BIXNIG	BIXNOM	CABSIH10	CASKAI
CECYIU	COKBIP	CUKHEW	DEJSAM	DOCYTC	DONBUD
DOURID	DOXPOV	DUHHUJ	DXCYTD	DXCYTD01	EHUFEU
ETYXUR	EWALAR	FESCIQ	FIHXID	FIQSEF	FMDURD02
FUWHEL	FUXBIJ	FUXBIJ01	GERGAL	GEXXIQ01	GIDTIW
GODTOI	GOQCAS	HAXRAB	HECMAG	HEVXOV	HEZWAL
HEZWEP	HEZWIT	HEZWOZ	HIFVEY	HOLSUY	IJIHIU
IJIHOA	IJIHUG	IPDXUR	IPDXUR01	JIBDIH	KAMCUW
KASVEF	LIJZIN	LOGYUC	MOLQUZ	MOLRAG	NEKBOV
POGGAT	PULVIB	QAJJES	QOKJUV	RAKLOG	REJHOG
REJJAU	REMSOR	RESMAD	RIDGOC	ROHHII	ROHHOO
SEDQEX	SERQUC	SERRAJ	SIFKEX	SOLLAH	SULZEF
THYDIN	THYDIN01	THYDIN02	THYDIN03	THYDIN04	THYDIN05
THYDIN06	THYMDN01	TUFFUV	TUFGEG	UFEWEJ	VAWNEO

VEXDOR	WAZZAA	WEVWIE	WISGEK	XAWYIF	ZAPWOC
ZASQUF	ZASRAM	ZOZVUF			

Table S3. PDB codes of high quality NDB structures ($d_{min} \leqslant 1$ Å and $R \leqslant 10\%$) used to compare sugar restraint targets compiled by Parkinson *et al.* and those proposed in this paper, against modeled bond distances and angles.

4RKV	4NLF	4RJ1	6NIZ	4QIO	4X1A	4MJ9	4HIF	4HIG	3P4J	1VTT	1O56
1KGK	1ICK	1D8G	352D	293D	292D	131D	1D48	2DCG	1DCG		

Table S4. PDB codes of the 617 nucleic acid structures with resolution between 1.0 and 3.0 Å, used to test the robustness of the proposed sugar restraints.

1CSL	1D4R	1D9H	1DC0	1DUH	1EI4	1EVV	1FQ2	1G4Q	1HQ7	1I0G	1I0J
1I0K	1I0M	1I0N	1I0P	1I2X	1I2Y	1I5W	1I9X	1IKK	1J6S	1JES	1JRN
1JZV	1KD3	1KD4	1KD5	1KGK	1KH6	1L1H	1L2X	1L3D	1L4J	1L6B	1LC4
1LNT	1M69	1MLX	1MSY	1NGT	1NLC	1NR8	1NTA	1NUJ	1NUV	1NVY	1NZG
1O0K	1P4Y	1P54	1PJG	1PJO	1PUY	1PWF	1Q93	1Q96	1Q9A	1QDA	1R3G
1S23	1S2R	1SA9	1T0D	1T0E	1UB8	1UE2	1VRO	1VZK	1WOE	1X9C	1XCS
1XJR	1Y26	1Y27	1Y3O	1Y3S	1Y6T	1Y7F	1Y84	1Y86	1Y8L	1Y8V	1Y99
1Y9F	1Y9S	1YB9	1YBC	1YLS	1YXP	1Z43	1Z7F	1Z7I	1ZCI	1ZEX	1ZEY
1ZF0	1ZF1	1ZF3	1ZF4	1ZF6	1ZF7	1ZF8	1ZF9	1ZFA	1ZFB	1ZFG	1ZFT
1ZFV	1ZFX	1ZPH	1ZPI	1ZZ5	2A04	2A43	2B0K	2B1D	2B2B	2B3E	2B57
2BCY	2BCZ	2D2K	2D2L	2EES	2EET	2EEU	2EEV	2EEW	2ESJ	2ET4	2ET5
2ET8	2FCX	2FCY	2FCZ	2FD0	2FGP	2FII	2FIJ	2FIL	2FQN	2G3S	2G9C
2GIS	2GRB	2GVR	2GW0	2GWQ	2GYX	2HBN	2HC7	2HOJ	2HOL	2HOM	2HOO
2I2I	2I5A	2NLM	2NPY	2O4F	2OBZ	2OE5	2OE8	2OEU	2OIJ	2OIY	2ORF
2ORH	2OUE	2P7D	2P7E	2P7F	2PKV	2PL4	2PLB	2PN4	2PWT	2Q1O	2Q1R
2QEF	2QEG	2R1S	2R20	2R21	2R22	2V7R	2VUQ	2XNW	2XO0	2YDH	2YGH
2ZY6	397D	398D	399D	3AJK	3B31	3B58	3B5A	3B5F	3B5S	3B91	3BBI
3BBK	3BBM	3BNL	3BNN	3BNO	3BNP	3BNQ	3BNR	3BNS	3BSE	3C3Z	3C44
3C5D	3C7R	3CDM	3CGP	3CGS	3CJZ	3CQS	3CR1	3DVV	3DVZ	3DW6	3DW7
3E5C	3E5F	3EY2	3EY3	3FAR	3FL6	3FO4	3FO6	3FT6	3FTM	3G4M	3GAO
3GER	3GES	3GM7	3GOG	3GSK	3GVN	3GX2	3GX3	3GX5	3GX7	3I5L	3IGT
3IQN	3IQP	3IQR	3K1V	3LA5	3LPV	3MJA	3ND3	3ND4	3NJ7	3NKB	3NYP
3NZ7	3OK2	3OPI	3OT0	3OZ3	3OZ4	3OZ5	3P4B	3P4C	3P59	3PA0	3Q50
3Q61	3QF8	3QRN	3QXR	3R1D	3S49	3S4P	3S7C	3S8U	3SD8	3SJ2	3SSF
3SYW	3SZX	3TD0	3TD1	3TVB	3U2N	3U38	3UKB	3UKC	3UKE	3UYB	3V06
3V07	3WRU	3ZD4	3ZD5	3ZP8	423D	424D	428D	429D	433D	437D	442D
443D	448D	449D	455D	462D	464D	466D	473D	476D	477D	480D	483D
4AH1	4C5X	4C63	4C64	4DKZ	4E58	4E59	4E7Y	4E8S	4E8X	4E95	4ENC
4F2X	4F2Y	4F3U	4F4N	4F8U	4F8V	4FE5	4FEJ	4FEL	4FEN	4FEO	4FEP
4FNJ	4FXM	4GJU	4GLC	4GLG	4GLH	4GRE	4HLI	4HQI	4I9V	4III	4IJ0
4ITD	4J50	4JAH	4JF2	4JRD	4K31	4KW0	4KWX	4KYY	4L25	4L26	4L81
4LTF	4LTG	4LTH	4LTI	4LTJ	4LTK	4LTL	4LX6	4LY2	4M3I	4M3V	4MCE
4MGW	4MKW	4MS5	4MSB	4MSR	4NFO	4NFP	4NFQ	4NLF	4NXH	4NYD	4O41
4O5W	4O5X	4O5Y	4O5Z	4OCD	4OKL	4P1D	4P20	4P3S	4P3T	4P3U	4P43
4P97	4PCJ	4PWM	4QC7	4QKK	4R44	4R45	4R49	4R4A	4R4D	4R8J	4RBQ
4RBY	4RBZ	4RC0	4RHD	4RNE	4RUM	4TS0	4TS2	4TZX	4U34	4U3O	4U3R
4U5M	4U6L	4U78	4U8A	4U8B	4U8C	4U92	4WO2	4X18	4XNO	4XQZ	4XW0
4XW7	4Y27	4YMC	5AXF	5AY2	5AY3	5AY4	5BJO	5BJP	5BWS	5C5W	5CCW
5CH0	5CJY	5D8T	5DA6	5DAM	5DHB	5DHC	5DO5	5DQK	5E36	5EMF	5ET2
5EW4	5EW7	5FHJ	5FJ0	5FJC	5FK1	5FK2	5FK3	5FK4	5FK6	5FKD	5FKE
5FKF	5FKH	5GSK	5HBW	5HBX	5HBY	5HN2	5HNJ	5HNQ	5IP8	5IU5	5IWJ
5IX7	5IYG	5IYJ	5JVW	5KRG	5KX9	5L00	5LFS	5LFX	5LIT	5LQT	5LR3
5LR4	5LS8	5MVK	5MVL	5MVP	5MVQ	5MWI	5NBE	5NDH	5NDI	5NEO	5NEP
5NEQ	5NEX	5NWQ	5NXT	5NY8	5NZ3	5NZ6	5NZD	5O62	5O69	5T3L	5T5A
5TDJ	5TDK	5TKO	5U0Q	5UED	5UEE	5UX3	5UZ6	5UZA	5V0H	5V0J	5V0K
5V0O	5V1K	5V1L	5V2H	5V9Z	5VCF	5VCI	5VJ9	5VJB	5VXQ	5W1Z	5W20
5WSQ	5WV7	5XK1	5XUV	5Z1H	5Z1I	5Z71	5ZAS	5ZAT	5ZEG	5ZEI	6A85
6ADV	6AU4	6BGB	6C8D	6C8E	6C8I	6C8J	6C8K	6C8L	6C8M	6C8N	6C8O
6CQ3	6CU1	6CXZ	6CY4	6DN3	6DY5	6E1S	6E1T	6E1U	6E1V	6E1W	6E81
6E84	6E8S	6E8U	6F3C	6FZ0	6G8S	6GIM	6H0R	6H5R	6HBT	6HBX	6HC5

6HU6	6IP3	6IUE	6IYQ	6JR4	6JV3	6MC2	6MC3	6N4G	6P2H	6QIQ	6QIR
6QIS	6QIT	6QIV	6QN3	6RNL							
